# Integrative Genomic Signatures Of Hepatocellular Carcinoma Derived from Nonalcoholic Fatty Liver Disease

**DOI:** 10.1371/journal.pone.0124544

**Published:** 2015-05-20

**Authors:** Itziar Frades, Erik Andreasson, Jose Maria Mato, Erik Alexandersson, Rune Matthiesen, Mª Luz Martínez-Chantar

**Affiliations:** 1 Metabolomics Unit, CIC bioGUNE, Centro de Investigación Cooperativa en Biociencias, Bizkaia Technology Park, Derio, Bizkaia, Spain; 2 Department of Plant Protection Biology, Swedish University of Agricultural Sciences, Alnarp, Sweden; 3 Department of Human genetics, National Health Institute Doutor Ricardo Jorge, Lisboa, Portugal; University of Navarra School of Medicine and Center for Applied Medical Research (CIMA), SPAIN

## Abstract

Nonalcoholic fatty liver disease (NAFLD) is a risk factor for Hepatocellular carcinoma (HCC), but he transition from NAFLD to HCC is poorly understood. Feature selection algorithms in human and genetically modified mice NAFLD and HCC microarray data were applied to generate signatures of NAFLD progression and HCC differential survival. These signatures were used to study the pathogenesis of NAFLD derived HCC and explore which subtypes of cancers that can be investigated using mouse models. Our findings show that: (I) HNF4 is a common potential transcription factor mediating the transcription of NAFLD progression genes (II) mice HCC derived from NAFLD co-cluster with a less aggressive human HCC subtype of differential prognosis and mixed etiology (III) the HCC survival signature is able to correctly classify 95% of the samples and gives Fgf20 and Tgfb1i1 as the most robust genes for prediction (IV) the expression values of genes composing the signature in an independent human HCC dataset revealed different HCC subtypes showing differences in survival time by a Logrank test. In summary, we present marker signatures for NAFLD derived HCC molecular pathogenesis both at the gene and pathway level.

## Introduction

Nonalcoholic fatty liver disease (NAFLD) is a condition where fat deposits in the liver. NAFLD refers to a wide spectrum of liver diseases such as fatty liver (steatosis) and inflammation derived nonalcoholic steatohepatitis (NASH). This condition can advance to fibrosis and cirrhosis producing a progressive, irreversible liver scarring that in the 15% of the cases progress into a liver hepatocellular carcinoma (HCC)[1]. The factors implicated in this progression are poorly understood.

NAFLD is believed to be the hepatic manifestation of the metabolic syndrome, which includes central obesity, insulin resistance, dyslipidemia and hypertension [1]. The two-hit hypothesis [2] states that in a first hit an imbalance in fatty acid metabolism occurs producing the hepatic triglyceride accumulation (steatosis). The second hit results from efforts to compensate for altered lipid homeostasis and consist of oxidative/metabolic stress and deregulated cytokine production. In addition, Jou et al. [1] have proposed a third fibroinflammatory repair hit due to overwhelmed hepatocyte survival mechanisms and increased hepatocyte death rates. This drives progression from NASH to cirrhosis as these regenerative responses activate the hepatic stellate cells to myofibroblasts that cause liver fibrosis. Regenerative responses are responsible for the expansion of the hepatic progenitor populations that produce chemoattractants to recruit various types of immune cells into the liver.

Steatosis and NASH develop as a result of excessive pro-inflammatory factors. The etiology of NASH has a necro-inflammatory component modulated by interactions among various factors that regulate the biological activity of TNFα. Faced with excessive TNFα and fatty acids hepatocytes store lipids and activate NF-κB within hepatocytes. Hepatocyte oxidative stress and eventual apoptosis is promoted by the local increase in TNFα which also recruits inflammatory cells from the immune system into the liver signifying the emergence of NASH [3]. In 25% of the cases there is a progression from NASH to cirrhosis where leptin inducible factors that regulate the activity of profibrogenic cytokines, such as TGF-β, dictate the extent of fibrosis that occurs during liver injury [3]. When tissue homeostasis is chronically perturbed, interactions between innate and adaptive immune cells can be disturbed. Then cells from the innate immune system immediately release soluble mediators, such as cytokines, chemokines, matrix remodeling proteases and reactive oxygen species. These are factors that induce mobilization and infiltration of additional leukocytes into damaged tissue resulting in a chronic inflammation [4]. This results in excessive tissue remodeling, loss of its architecture due to tissue destruction, protein and DNA alterations due to oxidative stress and under some circumstances, increased risk of cancer development [3]. See S1 Table in S1 File for a review of the most established biological processes and biomarkers for NAFLD.

HCC is the fifth most common cancer in the world. The variability in the prognosis of individuals with HCC suggests that HCC may comprise several distinct phenotypes [5]. These phenotypes may result from the activation of different oncogenic pathways during tumorigenesis as the development of an oncogenic state is a complex process involving the accumulation of multiple independent mutations that lead to deregulation of cell signaling pathways central to the control of cell growth and cell fate [6]. Hepatitis B (HBV), hepatitis C virus (HCV) [7], smoking [8], reproductive and hormonal factors [9], liver cirrhosis [10], primary biliary cirrhosis [11], diabetes [12], NAFLD [13] and the metabolic syndrome [14], alcohol intake [15] and overweight and obesity [16] are causes of HCC.

The glycine N-methyltransferase knockout (GNMT KO) [17] and the methionine adenosyl transferase knockout (MAT1A KO) mouse models develop NAFLD stages. These models have altered the *S*-adenosyl methionine (SAMe) production. SAMe is a cofactor involved in methyl group transfers, process which is involved in the epigenetic silencing of gene expression by methylating promoter regions [18]. The MAT1A KO suffer from lack of SAMe [19] while the GNMT KO has an excess of SAMe leading to aberrant methylation patterning of the DNA that results in liver disease phenotype [18].

For medical diagnostics, a major task is to find a set of genes correlated with given phenotypes designated as signatures [20]. These signatures may reveal insights to biological processes and may be used to classify new samples. Different genes may be present in different signatures when different training sets of samples and different statistical tools are used. This is because many genes have correlated expression, especially the genes involved in the same biological process [21]. Reproducibility in gene signatures identified in different datasets is rare [22]. Therefore a major challenge for application of gene expression profiling is stability of the signature.

Robust signatures can be found using feature selection techniques meaning a selection of a subset of features which values maximize the classification performance. Therefore feature selection is a combinatorial optimization problem used to reduce the dimensionality in classification tasks. Reducing the dimensionality of the data by deleting unsuitable attributes improves the speed and also the performance of the learning algorithms that ultimately will be used for classification. Feature selection process has two main steps: search and evaluation of subsets of features.

In this study, a representative set of feature selection methods were adapted and implemented for microarray data. In order to build the feature selection models we adapted different search strategies including sequential methods and intensive search algorithms such as those based on evolutionary approaches (S1-S3 Figs. in S1 File). We also used various kinds of supervised evaluation criteria based on induction algorithms and supervised clustering. Resampling techniques were used to assess both an approximately unbiased evaluation criteria and the stability of the feature selection models. This resulted in running the feature selection methods on different random partitions of the input data and then, an ensemble solution based on frequency aggregation of the resulting subsets was generated [23] in order to improve the stability while avoiding overfitting.

By applying these feature selection algorithms in human and genetically modified mice HCC and NAFLD two kinds of robust signatures in form of pathways and genes were defined. The first type, NAFLD progression signatures are common for human and mice and hold the mechanisms of disease progression. The second kind is a signature of HCC survival containing the molecular features that discriminate individuals of a poor from a good prognosis.

## Materials and Methods

### Samples, microarray platforms and GEO accession numbers

RNA samples for microarray experiments of GNMT KO mouse were extracted at 3 and 8 month time when they were histologically determined to develop NASH and HCC respectively and samples from MAT1A KO mouse are extracted at 3–8 and 15 month time when they develop steatosis, NASH and HCC. The mice samples were collected specifically for this study. Animals were treated humanely, and all procedures were in compliance with our institution’s guidelines for the use of laboratory animals. The condition of the animals was monitored daily. The animals were anesthetized with 4% of isofluorane and sacrificed by cervical dislocation at the time points indicated above. The liver was frozen and paraffin samples were collected to analyze the status of the liver. The health conditions of the mice were not compromised in this study. Gene expression microarray experiments were done on the Affymetrix GeneChip Mouse Genome 430 2.0 Array and 430A 2.0 Array.

Previously published human samples of steatosis and NASH were used [24]. These were hybridized with the Affymetrix HG-U133_Plus_2.na22 platform. Publicly available human HCC samples from the GEO GSE1898 [5] and GSE364 [25] series with the GPL1528 and GPL257 microarray platforms respectively were used. GSE1898 series has HCC samples for which survival data is available and these were integrated with NAFLD derived HCC from genetically modified mice to create signatures distinguishing HCC subtypes characterized because of having a different prognosis. The survival analysis is based solely on the publicly available human survival data. GSE364 dataset was used as a test set because human HCC survival data is also available. See Table 1 for an overview on the samples, microarray platforms and GEO accession numbers.

**Table 1:** Microarray samples (biological replicates), platforms and GEO accession numbers.

Microarray samples, platforms and GEO accession numbers		*Steatosis*		*NASH*			*HCC*	
		*Samples-biological replicates*	*Platforms*	*samples-biological replicates*		*Platforms*	*samples-biological replicates*	*Platforms and GEO accession numbers*
***Signatures of NAFLD progression***	***Mice***	-5 biological replicates of 3 month MAT1A KO mouse	Affymetrix Mouse430_2.na21 platform		-5 biological replicates of 3 month GNMT KO-5 biological replicates of 8 month MAT1A KO mouse	Affymetrix Mouse430_2.na21 platform	-4 biological replicates of 8 month GNMT KO-5 biological replicates of 15 month MAT1A KO mouse	Affymetrix Mouse430_2.na21 platform
	***human***				-9 human biological replicates	Affymetrix HG-U133_Plus_2.na22 platform		
***Survival signature***	***Mice***						-4 biological replicates of 8 month GNMT KO-5 biological replicates of 15 month MAT1A KO mouse	Affymetrix Mouse430_2.na21 platform
	***human training***						-91 human biological replicates	GPL1528 human microarray platform in GSE1898 series
	***human validation***						-87 human biological replicates	GPL257 human microarray platform in GSE364 series
***Differentially expressed genes in steatosis and NASH***	***human***	-2 human biological replicates	Affymetrix HG-U133_Plus_2.na22 platform		-9 human biological replicates	Affymetrix HG-U133_Plus_2.na22 platform		

We performed a RMA normalization where the log_2_ ratios (M values) of knockout versus wild type or disease vs control were calculated according to [26]. Probes belonging to the same genes were averaged.

The Institutional Animal Care and Use Committee (IACUC) that approved specifically this study were the Bioethical and Animal Welfare Committee of the CIC bioGUNE. Codes: Breeding of MAT1A: P CBG CBBA 1412. Breeding and expansion of GNMT KO: P CBG CBBA 1512. Characterization of mouse lines GNMT and MAT1A KO: P CBG CBBA 2010. The Institutional Review Board that approved this specific study using human samples was the Human Research Review Committee of the Hospital de Alcalá de Henares de Madrid. All subjects gave their signed consent to liver biopsy and to participate in this study.

### Feature selection methodologies

In order to carry out the signature based analysis, a versatile series of feature selection algorithms was adapted and implemented (Table 2). According to the search procedure the multivariate algorithms make use of a genetic algorithm (GA) (S3 Fig in S1 File) that uses an evolutionary approach which applies the evolutionary operators to guide the moves along the space of solutions, as well as three different heuristic sequential methods for feature selection. These include a backward multivariate method with recursive feature elimination (RFE) (S1 Fig in S1 File), a multivariate forward feature selection method called minimum redundancy maximum relevance (MRMR) (S2 Fig in S1 File) [27], an hybrid approach of these last two methods called recursive feature elimination minimum redundancy (RFE_MR) (S1 Fig in S1 File) and the knowledge-driven approaches of this last. Some of these knowledge-driven approaches minimize the correlation among the selected genes (RFE_MinR_MinGO). As a high degree of redundancy can suppose that two genes belong to the same pathway, are coexpressed or are on the same chromosome, other knowledge-driven approaches tackle the redundancy in opposite way, so they maximize correlation (REF_MaxR_MaxGO). The univariate search methods explained in [28] were also adapted resulting in forward feature selection search methods (GS1, GS2 and F-TEST).

**Table 2:** The 26 feature selection methods.

		Search strategies			
		Sequential			Evolutionary approach
		Backward Elimination		Forward feature selection	
**Evaluation criteria**	**Wrapper**	**-5 fold crossvalidation of the specified based classifier**	**RFE-SVM (nr,m)RFE-NB(nr,m)RFE-BN(nr,m)**		
	**Filter-wrapper hybrid**	**-Clustering-distance matrix**	**RFE (nr,m)RFE_MR(r,m)RFE_MinR_MinGO(r,m)REF_MaxR_MaxGo(r,m)**	**MRMR(r,m)**	**GA(nr,m)**
		**-Clustering-distance matrix-10 fold crossvalidation of SVM based classifier**		**GS1(nr, h)GS2(nr, h)F-TEST(nr, h)**	
		**-Clustering-External validity-Clustering choice:FOM**	**RFE_clust_FOM (nr,m)RFE_MR_clust_FOM (r, m)**	**MRMR_clust_FOM (r, m)GS1_clust_FOM (nr,h)GS2_clust_FOM(nr,h)F-TEST_clust_FOM(nr,h)**	**GA_clust_FOM (nr,m)**
		**-Clustering-External validity-Clustering choice:DUNN**	**RFE_clust_ DUNN (nr,m)RFE_MR_clust_ DUNN (r, m)**	**MRMR_clust_ DUNN (r, m)GS1_clust_ DUNN (nr,h)GS2_clust_ DUNN (nr,h)F-TEST_clust_ DUNN(nr,h)**	**GA_clust_ DUNN (nr,m)**

The methods are described in terms of the search and evaluation procedure they use, whether they tackle redundancy (r, redundant; nr, non-redundant), the name feature selection method and whether they are univariate (u), multivariate (m) or a hybrid of these two (h).

### The evaluation of the feature subset was done in three ways in all these search methods:

(1) Operating over the distance matrix that would be ultimately used by a hierarchical clustering algorithm to test the subset of selected features given the classification. The procedure relied on selecting the feature subsets that maximize the inter-cluster distance while minimize the intra-cluster distance using a predetermined classification. (2) Using three supervised induction algorithms to evaluate the selected subsets (Support Vector Machines and two configurations of Naïve Bayes). (3) Based on supervised clustering and external validation: at each iteration the output of an optimal unsupervised clustering algorithm among a representative set of clustering strategies is compared with the dataset’s real partitioning to evaluate the subsets of features. Instead of using a single classification method to perform the evaluation of the subsets, this evaluation procedure chooses the optimal method among a set of clustering procedures. The optimal method was chosen in two ways: the clustering algorithm maximizing the Dunn index (DUNN) or the clustering algorithm minimizing the Figure of Merit (FOM). The set of clustering algorithms include k-means, Diana, sota, pam, clara and average, complete, single and ward linkage criterion for hierarchical clustering and agnes.

Redundancy was measured as the gene average pairwise mutual information or as the average gene ontology (GO) term pairwise similarity in the selected gene subset. The inclusion of the gene GO term pairwise similarity as a redundancy measure to guide the search resulted in knowledge-driven feature selection methodologies (RFE_MinR_MinGO and REF_MaxR_MaxGO).

As recent advances include the development of therapies targeting specific signalling pathways, these feature selection methods were adapted for microarray analysis by classifying disease based not only on the activity of individual genes but also on the deregulated over-represented signalling pathways to obtain further biological insight. We identified KEGG pathway maps enriched in each of the subset of genes resulting from the five-fold crossvalidation procedure whose combined expression delivers optimal discriminative power for the class variable, obtaining the overrepresented deregulated pathways that distinguish the different conditions. These pathways are deregulated because it was applied a preprocessing step where only those genes that were deregulated in a 20% of samples were selected, while significant over representations of genes in functional categories were defined based on the hypergemetric test.

Linear lowpass filtering also called smoothing data of time series was applied as a preprocessing step where the expression values were decomposed into random variation, cyclic variation and trend component. This preprocessing step aimed at stabilizing the feature selection algorithms and consisted in using the trend component to feed the feature selection algorithms removing random and cyclic variation. This approach also tried to avoid over-fitting of the classifiers.

Two further approaches were taken to avoid overfitting: the use of both adequate evaluation criteria and stable and robust feature selection models. Resampling techniques were used to estimate the approximately unbiased classification performance and assess the robustness or stability of the feature selection technique, indicating how sensitive the output of a feature selection method is to random perturbations in the input data [29]–[30]. This made possible to define the stability of selected feature subsets, individual features (genes) and over-represented deregulated pathways.

**Fig 1:**
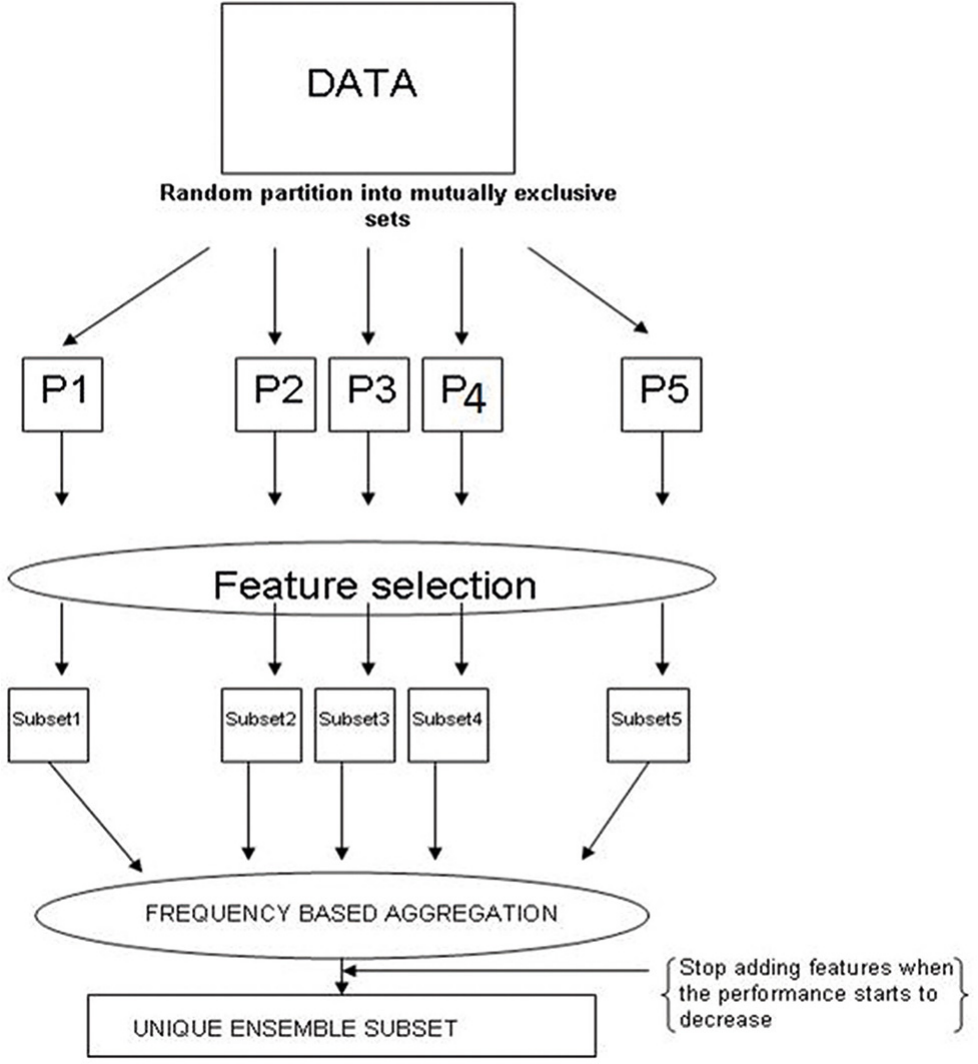
Data partition and aggregation procedures. A random partition of the data into mutually exclusive sets P_1_, P_2_, P_3_, P_4_ and P_5_ is done. Feature selection is performed in each partition. It results in a feature subset for each partition. We perform frequency based aggregation by individually adding the most frequent features from the subsets and stop adding features when the performance of a mining algorithm starts to decrease. It results in a unique ensemble subset.

Five-fold crossvalidation scheme was used because it preserves a reduced bias in comparison with resubstitution, it estimates the error with lower variance and uses less computational time compared to the leave-one-out crossvalidation [29]. The feature selection process is external in training the classification rule at each stage of the accuracy estimation procedure. It results in running the feature selection algorithm five times and recording the selected set of features on each run to introduce variability, this way ensuring that the feature selection algorithms start in different locations in the search space and choose different initial subsets to begin the search process from [23] (Fig 1).

To assess the stability of a feature selection technique, variation in the distribution of features present in the subsets selected under different partitioning of the training/input data was calculated. The measure used to assess the stability of the selected subsets was the Normalized Average Hamming distance (NAHD) [23, 31] between the five subsets resulting from the five-fold crossvalidation. NAHD measures the average of the minimum number of substitutions required to change one into the other. Another stability indicator is the frequency with which a given gene is selected across subsamples. The frequency of each of the deregulated KEGG pathways showing overrepresentation [32–34] as tested by the hypergeometric test for each of five runs of the selection algorithms was also recorded.

This analysis design where there are five runs of each of the different methods allowed to further explore the produced signatures in each of the algorithms in terms of their gene composition frequency and frequency of the enriched deregulated KEGG pathways. By selecting the minimum amount of genes and overrepresented KEGG pathway which expression patterns maximized the classification performance of the phenotypes in their corresponding classes, each of the feature selection runs in the external five-fold crossvalidation procedure produced a genomic signature of genes and another one of pathways. These expression signatures showed phenotype and sample discrimination capabilities. To provide more robust feature subsets it was made a solution to the instability of the feature selection method based on the frequency aggregation of the five subsets resulting from the five runs of the crossvalidation which is essentially an ensemble solution that can be called rank summation [23]. Finally the same frequency based aggregation procedure to combine the genomic signatures produced by the different methods to further maximize the classification performance and find unique convergent ensemble signatures was applied.

### Clustering analysis

Bootstrap resampling techniques were used to assess the uncertainty in hierarchical cluster analysis by calculating probability values (p-values) for each cluster in the dendrogram that represents the possibility that the cluster is the true cluster. Two types of p-values were available: bootstrap probability (BP) value and approximately unbiased (AU) p-value. In both cases thousands of bootstrap samples were generated by randomly sampling with replacement elements of the data and bootstrap replicates of the dendrogram were obtained by repeatedly applying cluster analysis to them. BP is biased as discussed in [35–39] and multiscale bootstrap resampling was used for the calculation of AUp-value [38, 40–42] which has superiority in bias over BP value calculated by the ordinary bootstrap resampling.

**Fig 2:**
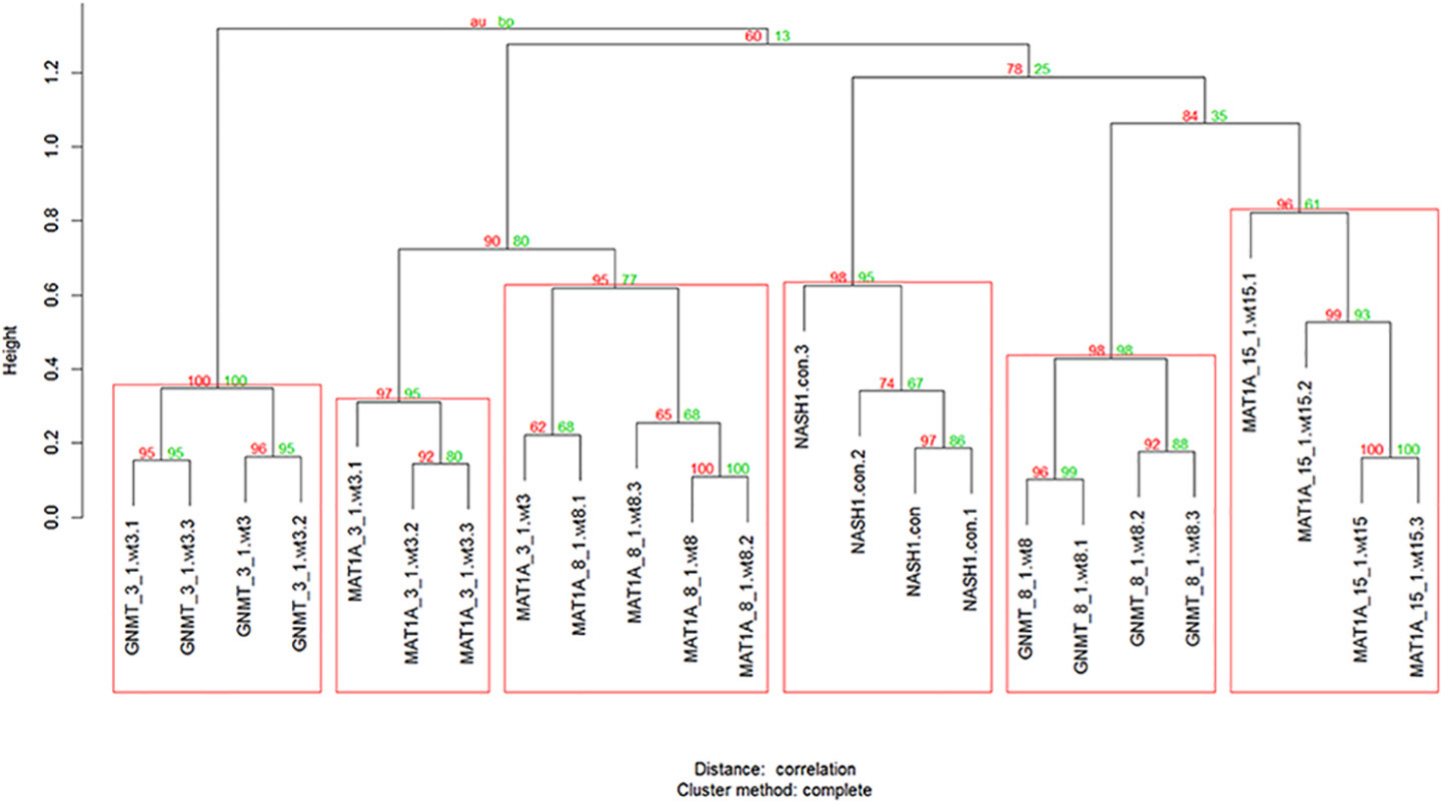
Tree structure where each of the stages of the disease has been clustered in a single cluster using the RFE_clust_Dunn algorithm to select the variables used as input in pvclust [43] used to perform hierarchical clustering.

**Fig 3:**
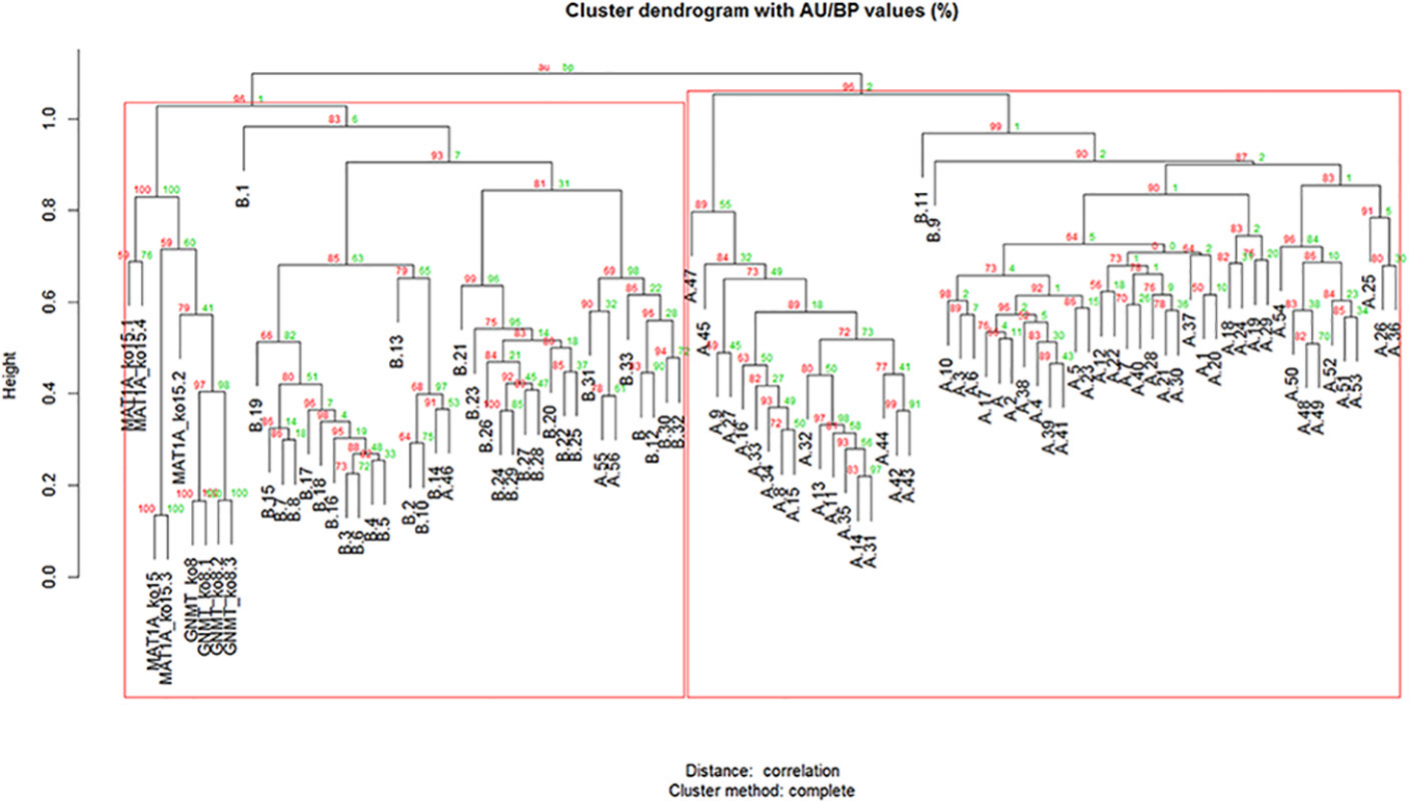
Mouse and human HCC clustering. the gene expression data of the human HCC of mixed etiologies has been integrated with HCC samples from GNMT and MAT1A mouse KO models of HCC derived from NAFLD by selecting the orthologous genes using the homologene database. The integrated data holds 1691 genes obtained from matching the orthologous genes between the genes having at least 9 samples of two fold regulation in the human HCC series, the 15 month MAT1A KO and 8 month GNMT mouse KO models. Using complete hierarchical clustering and Pearson correlation it is possible to distinguish cluster A and B with significant differences of survival length and the mouse models laying together cluster A.

**Fig 4:**
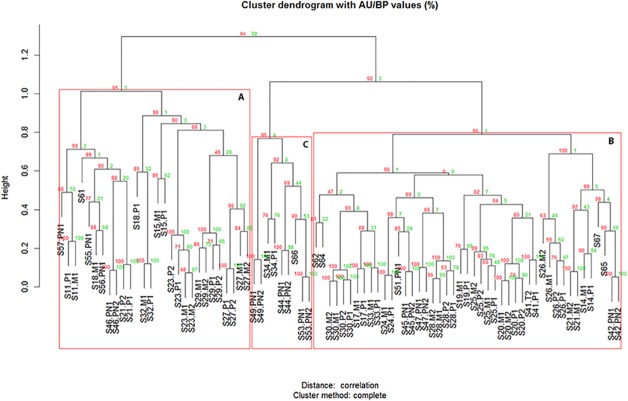
Survival signature common for human and mouse in an independent HCC dataset using complete hierarchical clustering and Pearson correlation as a similarity measure over the expression values of the genes composing renders 3 main clusters (A, C and B) representing HCC subtypes of differential survival.

Clusters with AU larger than 95% were highlighted by rectangles, which are strongly supported by data as in a cluster with AU p-value > 95%, the hypothesis that "the cluster does not exist" is rejected with significance level 0.05 (Figs 2, 3 and 4 & S5 and S6 Figs. In S1 Fie).

### Signatures of NAFLD progression

For the signatures of NAFLD progression microarray samples from different stages of the disease from human and mouse were collected to perform a time course analysis. Using a battery of 14 newly adapted feature selection approaches (Table 3) robust signatures of NAFLD progression were defined. Mouse and human data was integrated by selecting the orthologous genes using homologene database [44]. Only genes having twofold regulation or more in 20% of the samples were selected (470 genes).

**Table 3:** 5 fold cross-validation classification performance, stability calculated as the Average Normalized Hamming Distance (ANHD) and number of selected genes in the signatures of NAFLD progression from smoothed and raw data.

Method	5 fold crossvalidation classification performance smoothed data	5 fold crossvalidation classification performance raw data	Genes smoothed data	Genes smoothed data	ANHD smoothed data	ANHD raw data	Ensemble error smoothed data	Ensemble error raw data
**GS1**	0.065±0.009	0.084±0.016	28	39	0	6.577	0.08	0.092
**GS2**	0.070±0.010	0.087±0.019	39	39	0	8.156	0.061	0.093
**F-TEST**	0.077±0.012	0.086±0.019	43	54	0	8.020	0.054	0.095
**RFE**	0.033±0.015	0.043±0.011	28	61	0	3.955	0.054	0.067
**RFE_MR**	0.067±0.009	0.085±0.020	50	373	0	5.065	0.061	0.093
**RFE_SVM**	0.135±0.048	0.232±0.130	11	26	0	0.756	0.144	0.091
**RFE_BN**	0.042±0.044	0.072±0.036	58	84	0	5.678	0.064	0.101
**RFE_NB**	0.217±0.082	0.217±0.061	49	70	0	3.152	0.054	0.051
**GA**	0.027±0.009	0.042±0.007	111	67	0	5.665	0.058	0.058
**MRMR**	0.060±0.020	0.076±0.015	35	371	0	5.140	0.08	0.097
**RFE_MinR_MinGO**	0.070±0.014	0.090±0.021	50	85	0	4.582	0.067	0.092
**REF_MaxR_MaxGo**	0.068±0.026	0.088±0.017	218	93	0	5.658	0.077	0.085

Initially raw expression values were used for the 14 supervised clustering feature selection algorithms (Table 3). Then, to cancel the effect due to random variation and stabilize the algorithms, weighted moving averages a kind of linear lowpass filtering preprocessing was applied (Table 3). Four samples of each of the human and mouse disease stages representing the progressive NAFLD stages were used as a smoothing parameter. These time course profiles were treated as time series and the raw data were replaced by the trend component to feed the feature selection procedures. To generate more robust solutions the signatures produced by the different methods were aggregated by rank summation.

For the genes composing these signatures enrichment of transcription factor binding sites were explored by the OPPOSUM program using a Fisher exact test (p<0.05) [45] (S1 Table in S1 File). The positional gene enrichment analysis using PGE program [46] was used to explore co-localisation of genes in the signatures in the same chromosome band (S4 Fig in S1 File).

### Survival signature for human and mouse

By selecting the orthologous genes using homologene database [44] the mouse KO HCC models data were integrated with 91 samples of human HCC gene expression data [5] where clustering analysis previously revealed two HCC subtypes A and B in the prognosis of the individuals resulting from the activation of different tumorigenic pathways (S5 Fig in S1 File). Clustering analysis of the integrated human and mouse microarray data again showed the two cancer subtypes A and B (Fig 4). Using feature selection methodologies gene expression signatures derived from integrated data were generated. To construct the unique common survival signature for human and mouse 297 differentially expressed genes (DEGs) between cluster A and B were identified (two sample t test; p<0.001). This list of genes was then introduced in five of the feature selection methodologies (GS1, GS2, F-TEST, RFE_SVM and MRMR) (S3 Table in S1 File) and by rank summation [23] of the signatures a unique signature was obtained.

Venn diagrams were defined to capture genes that were differentially expressed in human steatosis and NASH when comparing cases and controls (two-sample t-test; p<0.05). Then we identified the common genes in the prognostic signature present between the differentially expressed genes in human steatosis and NASH.

The signature holding the genes determining statistical differences in survival length was further validated by a Logrank test with an independent human HCC dataset having a HBV etiology and for which survival data was available [25]. Hierarchical clustering analysis on the expression values of the genes composing the signature in the independent dataset was applied to define different HCC subtypes (Fig 4). Survival analysis was done to check whether there were statistical differences in survival length among the subtypes by Logrank test and Kaplan-Meier plots (Fig 6).

## Results and Discussion

In this study a series of newly adapted feature selection approaches was used to define different robust signatures holding the pathways and genes involved in NAFLD progression as well as a signature of differential survival in HCC common for human and mouse.

### Signatures of NAFLD progression hold convergent pathways regulated by HNF

The NAFLD progression signatures were used to study the pathogenesis of NAFLD derived HCC. Gene expression and pathway deregulation signatures with the genes and pathways that can distinguish different disease stages in human and mice were found using as input 471 genes having twofold regulation or more in 20% of the samples. The signatures produced by the 14 supervised clustering feature selection algorithms described in material and methods were aggregated to generate more robust solutions (Table 3). Weighted moving averages linear lowpass filtering was also applied to remove random variation from the data and run the feature selection algorithms. The performance, stability and variance of the feature selection procedures developed using raw data and smoothed data were compared (Table 3). It was observed that filtering the expression profiles using weighted moving averages produced a huge positive impact on the stability of all the feature selection methodologies as NAHD was reduced to 0. This preprocessing step also reduced the variance (Table 3).

Using this preprocessed data the 14 supervised clustering and external validation feature selection algorithms were run in order to find signatures which produce the optimal cluster (Fig 2). Both the RFE_clust_Dunn, that maximizes the Dunn index and the GS1_clust_FOM that minimizes the FOM are examples of stable optimal clusters (Fig 2 & S6 Fig in S1 File & S4 Table in S1 File).

**Fig 5:**
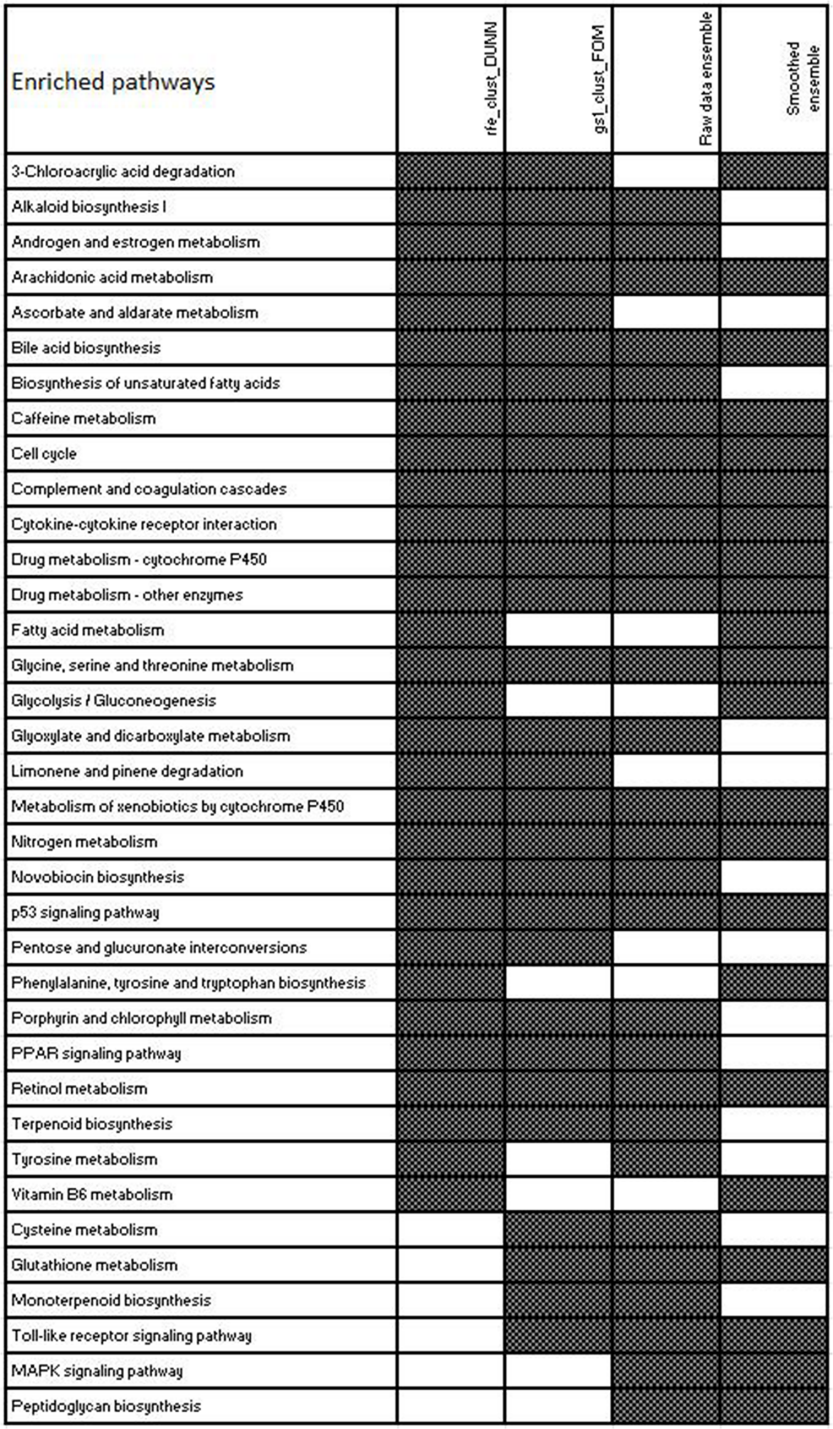
Enriched KEGG pathway signatures selected by the two supervised clustering based feature selection methods which produced the optimal clustering result on smoothed data and the two ensemble signatures derived from 14 feature selection algorithm from raw and smoothed data used to build the signatures of NAFLD progression. KEGG enrichment analysis was performed on the genes selected in the 5 feature selection runs of the external 5 fold crossvalidation procedure and those pathways having a significant p-value (p<0.05) were selected.

The signatures produced by the 14 supervised feature selection methods for raw and smoothed data were aggregated separately and then the resulting pathway signatures were compared with the two feature selection methods which produced the optimal clustering result. It was possible to investigate the functional convergence in these signatures in terms of the overrepresented deregulated pathways whose expression levels can distinguish different disease stages and it was found that the different methods converge in similar functional solutions (Fig 5). The activity of a signaling pathway may currently be best characterized by the expression levels of its target genes. This approach is a simplification as it omits the fact that the expression levels of the components of the pathway are not necessarily affected when a pathway is activated. For example, a mutation or post translational modification of a transcription factor can change the expression levels of its target genes, without affecting the expression levels of the transcription factor itself or other components of the pathway. Below follows a description of the mechanisms that were found to be disregulated (S11-S23 Figs. in S1 File & Fig 5) and to contribute to disease progression in NAFLD [1]:

### Implications of the adipose tissue in NAFLD pathogenesis and progression

Hepatocyte accumulation of triglyceride is known to be a key component in the development of steatosis and NASH. Hepatic steatosis results from abnormal hepatocyte lipid metabolism that was found to be altered along the disease progression (Fig 5). Triglyceride storage itself is not hepatotoxic, but it is a marker of increased exposure of hepatocytes to potentially toxic fatty acids. Within this context stearoyl-CoA desaturase that catalyzes a rate-limiting step in the synthesis of unsaturated fatty acids was found to be upregulated in human steatosis, NASH and 3 month mouse KO models while downregulated in 8 month mouse KO models and with members up and downregulated in 15 month KO mouse model (S11 Fig in S1 File & S12 Fig in S1 File). It was also found that tyrosine metabolism in human steatosis and NASH samples as well as in the 8 month GNMT KO mouse model was downregulated (S13 Fig in S1 File). This metabolism ends by liberation of acetoacetate that can be connected to lipid synthesis. It has been hypothesized that altered unsaturated fatty acids could mediate PTEN down-regulation which triggers hepatic steatosis via an NF-kappaBp65/mTOR-dependent mechanism [47]. Our study also suggests the importance of androgen and estrogen metabolism central to fat accumulation and the metabolic syndrome as the pathways of these hormones have different members up and downregulated in human samples, downregulated in GNMT KO and upregulated in MAT1A KO (S14 Fig in S1 File).

#### Lipotoxicity and progression from steatosis to NASH

The arachidonic acid metabolism was upregulated in all mouse KO HCC samples and in all human samples (S15 Fig in S1 File). This metabolism is implicated in formation of prostanoids (Fig 5). The cyclooxygenase 2 (COX-2) catalyzes their rate-limiting step (S16 Fig in S1 File). Hepatocyte COX-2 facilitates the development of steatohepatitis and this effect may involve PPAR alpha as COX-2 increases the accumulation of PPAR alpha which promotes fatty acid β-oxidation [48]. PPAR signalling pathway was upregulated along the disease progression in human samples while decreases in mice (S17 Fig in S1 File). PPARγ activates pathways leading to fatty acid uptake (via CD36) and triglyceride synthesis [49–51]. The retinol metabolism was altered and hepatic loss of retinoic acids function leads to the development of steatohepatitis and liver tumors as retinoids downregulate the enzymes that are involved in mitochondrial β-oxidation of fatty acids while in contrast, upregulate the enzymes that are involved in peroxisomal β-oxidation [52]. Therefore it is possible to suggest that oxidative stress, depletion of hepatic long-chain polyunsaturated fatty acids and accumulation of excessive fat in the liver may underlie the pathophysiology of non-alcoholic steatohepatitis [53]. A consequence of this might be the observed general upregulation of P450s in fat-overloaded hepatocytes (S18 Fig in S1 File) [54], as well as impairment in the glutathione metabolism which is implicated in redox and detoxification [55]. P450 cytochromes are upregulated in both mouse KO models and human steatosis and NASH samples and HNF4a is responsible for the constitutive activity of the major P450 cytochromes in human liver [56]. It is also possible that NASH alters the xenobiotic activation of transcription factors that are known to induce the expression of cytochromes as this pathway is upregulated in human steatosis and HCCs from both mouse KO models (S19 Fig in S1 File) [57].

#### Implications of gut bacteria in the development of NASH

An upregulation of the Toll-like receptors along the disease progression was detected (S20 Fig in S1 File). This might be a consequence of the activation of the innate immune system response which receptors are typically Toll-like receptors. Alcohol has been shown to increase gut-derived lipoprotein component of endotoxin levels from Gram-negative bacteria as well as Gram-positive organisms in portal blood, activating toll-like receptors during alcohol-induced liver injury. Toll-like receptor activation may also play a role in steatohepatitis [58].

#### Possible insulin resistance in NAFLD

Both obesity and NAFLD are strongly associated with insulin resistance and hyperinsulinemia. Insulin is a key hormone in regulating lipogenesis and lipolysis in adipose depots. Insulin resistance in peripheral adipose tissues enhances lipolysis and increases delivery of adipose-derived fatty acids to the liver. We suggest that insulin resistance upregulates the renin-angiotensin system deregulated in our study (Fig 5) [59] decreasing energy expenditure, with augmented fat mass and affecting glucose clearance [60].

#### Linking hepatocellular carcinoma (HCC) with NAFLD

Hepatocyte DNA damage has been demonstrated in early stages of steatosis/NASH increasing formation of reactive oxygen intermediates that might damage DNA, overexpress p53 and increase Fas expression by hepatocytes [61]. This has been shown to increase with liver injury in animal models. Interestingly, we found p53 pathways to be upregulated in the progression of NAFLD (S21 Fig in S1 File). This finding suggests that steatosis/NASH provides a favourable ground for malignant transformation.

Related to the mechanisms involved in the progression of HCC in our animal models Clade B ovalbumin serpins (SERPINB1: serpin peptidase inhibitor, member 1. SERPINB6: serpin peptidase inhibitor, member 6. SERPINB9 serpin peptidase inhibitor, member 9) were upregulated in all HCC cases (S4 Fig in S1 File) and it has been reported that tumor is evaded against immunosurveillance by transcriptionally upregulating proteinase inhibitors which prevents the immune system from destroying the cancer cells [62].

Many mechanisms that might counteract malignant transformation (Fig 5) are found: MAPK pathways were upregulated in this study, such as Jun N-terminal kinase and p38 (S22 Fig in S1 File). The activation of p38 MAPK results in cancer cell apoptosis known to be initiated by retinoids, cisplatin and other chemotherapeutic agents [63]. Hepatic injury might be improved by bile acids which are also upregulated in 3 month mouse KO models, downregulated in 8 month ones and up and downregulated in 15 month MAT1A KO (S23 Fig in S1 File). It has been shown that they induce apoptosis in HepG2 cells [64]. Genes affecting monoterpenoids, also deregulated along the disease progression, are known to induce apoptosis in liver tumors [65]. Lastly α-linolenic acid was found to be deregulated along the disease progression. It is known to reduce COX-2 expression and to induce apoptosis of hepatoma cells [66].

The regulation of the expression of the genes in the signatures is often governed by transcription factors. Therefore the enriched transcription binding sites among the genes in the signatures factors were explored and HNF4 alpha was found to be the common transcription factor mediating the transcription of the genes composing the signatures (S1 Table in S1 File). This gene controls the development and metabolic homeostasis of the organism [67]-[68] and in agreement with previous studies the HCCs derived from two mouse KO models showed a strong downregulation of HNF4 [69, 70] (S7 Fig in S1 File). In human, HNF4 alpha was found to be downregulated in some NASH cases (S8 Fig in S1 File). HNF4 alpha is a zinc-coordinating group transcription factor. Overall, in mice NAFLD progression genes having transcription factor binding sites enriching HNF4 alpha are regulated in opposite direction (S7-S10 Figs. in S1 File). Examples of single zinc-sensitive transcription factors regulating gene expression in opposite directions exist [71].

Most of the other transcription factors controlling the expression of the genes in the different signatures (S2 Table in S1 File) are associated with liver disease where they are thought to play a role in the development of HCC. For example, the *nk-2*-related transcription factor is associated with human fetal liver and HCC since Nkx2.5 is involved in alpha fetoprotein transcription in HCC [72]. The proto-oncogenes c-fos is involved in cell cycle progression and cellular proliferation [61]. The gene has been associated to adenocarcinoma, carcinoma and liver neoplasms, DNA methylation and inflammatory response. The encoded protein by the Cebpa gene has been shown to bind to the leptin promoter and modulate its expression. The Cebpa protein can interact with CDK2 and CDK4, and thereby inhibiting these kinases and causing growth arrest. Functionally, the gene is associated with carcinoma and HCC. Pdx1 is associated to diabetes mellitus, glucose intolerance and early onset of diabetes in young people. The proto-oncoprotein Gfi regulates of SOCS gene expression by Gfi-1B inducing STAT5-target gene.

### HCC prognosis markers in the survival gene expression signature for human and mouse is validated by a test dataset

#### Identification of the two subtypes of HCC

HCC derived from genetically modified mice that spontaneously develop HCC arising from NAFLD was integrated with human HCC of different etiologies. This made it possible to investigate in what extent the mouse models reproduced features observed in the corresponding human conditions, as well as to understand the common molecular mechanisms between human HCCs derived from mixed etiologies and genetically modified mice HCCs derived from NAFLD. Unsupervised clustering procedures discriminated human microarray HCC samples of a less aggressive from a more aggressive phenotype and the mouse models co-clustered with the less aggressive of these HCC subtypes, possibly because mouse KO models do not develop metastasis (Fig 4 & S6 Fig in S1 File). Then, using different feature selection methods gene expression signatures were generated. These reflect the differential deregulation of biomarker genes and cell signaling pathways between the previously published two molecular HCC prognostic subtypes [5] with the integrated human and mouse microarray data (S3 Table in S1 File). Lastly a unique ensemble survival signature was generated by rank summation of the signatures produced by the different methods (Table 4). This signature was used to characterize and explore which cancers can be investigated using NAFLD derived HCC from genetically modified mouse models.

**Table 4:** Ensemble unique gene survival signature common for human and mouse resulting from the frequency based aggregation of the signatures produced by the 5 feature selection methods.

Gene ID	Gene name	Frequency
Tgfb1i1	**transforming growth factor beta 1 induced transcript 1**	1
Fgf20	**fibroblast growth factor 20**	1
Kcnk2	**potassium channel, subfamily K, member 2**	0.8
Pfkfb2	**6-phosphofructo-2-kinase/fructose-2,6-biphosphatase 2**	0.8
Kcnk3	**potassium channel, subfamily K, member 3**	0.8
Pigr	**polymeric immunoglobulin receptor**	0.8
Egr4	**early growth response 4**	0.8
Kera	**keratocan**	0.8
Foxf2	**forkhead box F2**	0.8
Adprh	**ADP-ribosylarginine hydrolase**	0.4
Cecr6	**cat eye syndrome chromosome region, candidate 6 homolog**	0.2
Slco1b2	**carrier organic anion transporter family, member 1b2**	0.2
Slc5a6	**solute carrier family 5 (sodium-dependent vitamin transporter), member 6**	0.2
Xkr4	**X Kell blood group precursor related family member 4.**	0.2
Camk1g	**calcium/calmodulin-dependent protein kinase I gamma**	0.2
Brd7	**bromodomain containing 7**	0.2
Mdfic	**MyoD family inhibitor domain containing**	0.2
D3Bwg0562e	**DNA segment, Chr 3, Brigham & Women's Genetics 0562 expressed**	0.2
Tnfsf13b	**tumor necrosis factor (ligand) superfamily, member 13b**	0.2
Muc13	**mucin 13, epithelial transmembrane**	0.2
Elf1	**E74-like factor 1 and similar to claspin homolog**	0.2
Ube2g2	**ubiquitin-conjugating enzyme E2G 2**	0.2
Ddx46	**DEAD (Asp-Glu-Ala-Asp) box polypeptide 46**	0.2

The frequency of appearance of the selected genes among the 5 feature selection methods is recorded as a measure of stability.

#### Performance of the unique survival signature for human and mouse

The resulting ensemble survival signature for human and mouse has an accurate classification performance and is quite stable as it classifies correctly 95% of the instances with a NAHD of 0.773. The frequency based aggregation procedure used to generate the ensemble solution produced a strong positive impact on stability and performance (S3 Table in S1 File).

#### Gene composition stability of the ensemble signature for human and mouse

The resulting gene subset composing the ensemble survival signature can be used as the prognostic markers that explain the differences in gene expression values among these two phenotypes of HCC. Here we described the most interesting patterns that classified the two molecular HCC subtypes (Table 4). There was great degree of functional convergence of pathways in the survival signature common for human and mouse resulting from pathways enrichment analysis of each of the 25 signatures produced by the five runs of the five feature selection methods (Table 5). The most stable genes for prediction in the ensemble signature were the fibroblast growth factor 20 (Fgf20) and the transforming growth factor beta 1 induced transcript 1 (Tgfb1i1). Fgf20 controls the extent of angiogenesis in liver disease [73]. Tgfb1i1 was upregulated in the cluster A and downregulated in cluster B. It is involved in the development of fibrosis in various processes of chronic inflammation in liver. It can promote invasion and metastasis during the tumor growth [74]. Other genes in the signature were 6-phosphofructo-2-kinase/fructose-2,6-biphosphatase 2 (Pfkfb2), Ubiquitin-conjugating enzyme E2G 2 (Ube2g2) and tumor necrosis factor ligand superfamily, member 13b (Tnfsf13b). The Pfkfb2 protein is involved in both the synthesis and degradation of fructose-2,6-bisphosphate, a regulatory molecule that controls glycolysis in eukaryotes. It induces glycolysis and is altered in both clusters. It can be speculated that this mutation in tumor cells can increase the energy-level of the fast growing tumor cells. Alterations in this pathway would also cause surface membrane alteration such as a decrease in glycoproteins that are important parts of adhesion molecules, in which abnormalities can result in acquisition of the metastatic phenotype. Supporting this, a positional gene enrichment analysis for the identification of chromosomal regions that were significantly enriched in the ensemble gene subset found 40% of enrichment in the q31 region of chromosome 1 that holds 6-phosphofructo-2-kinase and polymeric immunoglobulin receptor. Ube2g2 was downregulated in cluster A and upregulated in cluster B. These differences might explain the extent of apoptosis, which is limiting the proliferation and the accumulation of genetic and epigenetic alterations in the tumor, meaning that cancer cells have found a selective advantage in altering the apoptosis controlling enzymes [5]. Tnfsf13b acts as a potent B cell activator. This protein may be able to induce apoptosis through its interaction with other TNF receptor family proteins. TNF-induced cell death plays only a minor role compared to its overwhelming functions in the inflammatory process. Tnfsf13b was downregulated in cluster B while it was upregulated in cluster A. We only identified Tnfsf13b as a differentially expressed gene in steatosis and NASH together of being responsible of activating different tumorigenic pathways leading to differential survival of the individuals with HCC.

**Table 5:** Survival signature of pathways common for human and mouse resulting from the signatures produced by the 5 runs of the 5 feature selection methods.

Enriched KEGG pathways	Hypergeometric tests p-value	Standard deviation of p-value	Frequency
Regulation of autophagy	0.0103	0.0076	7
Reductive carboxylate cycle (CO2 fixation)	0.0257	0.0029	6
Neuroactive ligand-receptor interaction	0.0260	0.0020	4
Hematopoietic cell lineage	0.0272	0.0077	4
Folate biosynthesis	0.0382	0.0138	2
Starch and sucrose metabolism	0.0204	0.0109	2
Leukocyte transendothelial migration	0.0145	0.0028	2
Cell adhesion molecules (CAMs)	0.0081	0.0040	2

The frequency of appearance of the selected pathways among the 5 runs of the 5 feature selection methods is recorded as a measure of stability. Another measure of stability is the Hypergeometric test´s p-values standard deviation.

#### Validation of the survival signature using an independent dataset

**Fig 6:**
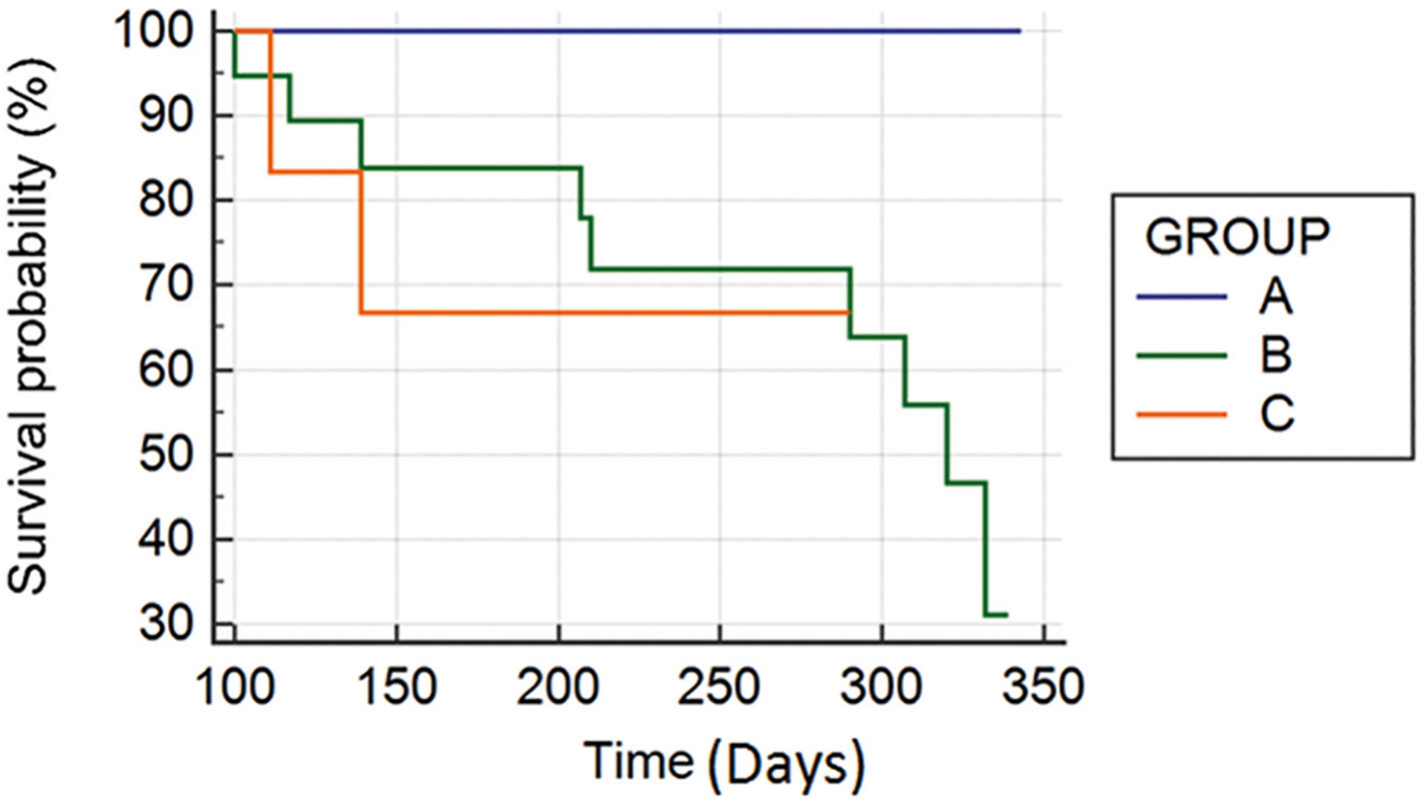
Kaplan-Meier plots showing the survival probability over time (days) of the 3 main clusters representing HCC subtypes of differential survival found in the independent HCC dataset when performing clustering analysis over the expression values of the genes composing the survival signature common for human and mouse.

The survival signature is composed of the common genes between human HCC from mixed etiologies and mouse HCCs derived from NAFLD that correlate with a differential prognosis. This is because it was built using feature selection methods to find the genes which the expression values discriminated between both HCC subtypes. The mixed etiologies from which the human HCCs are derived in the majority of the cases are HBV, HCV and alcohol. This survival signature was validated with an independent human HCC dataset with a HBV etiology having 87 samples [25]. Hierarchical clustering analysis was performed on the expression values of the gene families composing the signature and three different HCC subtypes were found (Fig 5) having statistical differences in survival length by Logrank test (p = 0,05) and Kaplan-Meier plots (Fig 6). Thus the survival signature was validated for discriminating between HCC prognostic subtypes of different etiologies including HBV, HCV, alcohol and NAFLD, as it was able to predict in an independent HCC different prognostic subtypes showing significant differences in survival time.

## Conclusion

From signatures generated by feature selection algorithms markers of HCC and targets for mechanistic studies were identified. These provide new insights into the molecular pathogenesis of NAFLD derived HCC. First the signatures of NAFLD progression common for human and genetically modified mouse models were generated to identify many of the known mechanisms of NAFLD progression. Most of the signatures have HNF4 as a common transcription factor controlling the transcription of their genes. Second, NAFLD derived HCC from genetically modified mouse models were integrated with human HCCs of mixed etiologies where previously unsupervised classification revealed two prognostic subtypes. The mouse HCC co-clustered with the less aggressive subtype. Possibly this is because the mouse KO models do not develop metastasis, which is the main feature of aggressiveness in a tumor. Indeed the most robust genes for prediction of prognostic subtypes are Fgf20 and Tgfb1i1 involved in angiogenesis, invasion and metastasis. HCC differential survival signature common for human and mouse was developed to allow for reliable identification of tumor type based on gene expression. This showed prognostic discrimination and prediction capabilities and reflected the differential deregulation of signaling pathways and genes between two molecular HCC prognostic subtypes. Importantly, the survival discrimination capabilities of the signature were validated with an independent human HCC dataset of HBV etiology. Cluster analysis on the expression values of the genes composing the signature in the independent human HCC dataset revealed different HCC subtypes showing differences in survival length, meaning that the survival signature can be extrapolated to other datasets of HCC with different etiologies. Improving the classification of individuals with HCC coming from diverse etiologies would create a basis for improving therapeutic strategies. Additionally, finding common processes between mice HCC derived from the progression of NAFLD stages and human HCC arising from diverse etiologies support the use of mouse models. Overall this analysis of data obtained from mouse models provide novel insight into the early development of HCC and point to novel therapeutic options.

## Supporting Information

S1 File S1 Fig. **Flow diagram of the steps performed by the RFE and RFE_MR method.** Stage1: As it is a backwards procedure starts from the full matrix of selected genes. The process is iterative where the number of iterations either for first selection (x = number of selection iterations) or posterior refinement selection around the selection solution (y = number of refinement iterations) should be specified. It uses the class vector as input. Stage2: Evaluate the selected gene subset. Stage3a. If the process does not take into account the redundancy of the features (RFE): calculates the sample by sample MI excluding each gene. For each excluded gene defines a coefficient I as the difference of the sum of the sample by sample MI between classes and the sum of the sample by sample MI within groups. Stage 3b1: If the process takes into account the redundancy of the features (RFE_MR): for each gene calculates the average gene pairwise mutual information. Stage3b2: For each gene calculates the Coefficient II value by adding the average gene pairwise MI to the coefficient II. Stage4: Remove the m worst coefficient values and their corresponding genes and expression values. Stage5: Find the minimum error rate along the iterations and get the selected genes. **S2 Fig. Flow diagram of the steps performed by the MRMR method.** Stage1: As it is a forward search procedure, it starts from an empty set of selected genes. The process is iterative where the number of iterations should be specified and uses the class vector as input. Stage2: Calculate the normalized mutual information of the class vector with the vector containing each gene expression values along the samples. Stage3: a. For each gene calculate the average gene pairwise mutual information. b. For each gene in the subset of selected genes calculate the average gene pairwise mutual information. Stage4: For each gene define a coefficient value by dividing the value of the normalized mutual information with the average gene pairwise mutual information. Stage5: Store the gene having the maximum coefficient value and remove from the matrix the corresponding gene. Stage6: Evaluate. Stage7: Find the minimum error rate along the iterations and get selected genes. **S3 Fig. Flow diagram of the GA procedure.** Stage 1: The procedure initially creates a number of random variable sets (chromosomes). These variable sets form a population of chromosomes. Each random set is created with an initialization that randomly selects 70 genes from the total 504. Stage 2: Each chromosome in the population is evaluated for its ability to predict the group membership of each sample in the dataset (fitness function). Stage 3: Elitism: select the fittest individual intact for the next generation. Stage 4: The population of chromosomes is replicated. The roulette wheel selection ensures that chromosomes with a higher fitness score will generate a more numerous offspring. Stage 5: The genetic information contained in the replicated parent chromosomes is combined through genetic crossover with a crossover probability (For the parameters see supplementary Table 4 and “*Parameters in the Genetic Algorithm*” supplementary section). The chromosomes are ranked according to their fitness value. Above the crossover probability the best chromosomes are maintained intact for the next generation. Below the crossover probability two randomly selected parent chromosomes are used to create two new chromosomes. This crossover mechanism allows a better exploration of possible solutions recombining good chromosomes. Stage 6: Mutations are then introduced in the new chromosomes generated by crossover randomly with a mutation probability. These mutations produce that new genes are used in chromosomes. Stage 7: The process is repeated from stage 2 until the number of generations exceeds certain threshold (100) and the regression between the population of chromosome’s minimum error rate and the generation is less than 0.05. The cycle of replication (stage 3), genetic crossover (stage 4) and mutations (stage 5) is called generation. **S4 Fig. Tree structure where each of the stages of the disease has been clustered in a single cluster.** Tree structure where each of the stages of the disease has been clustered in a single cluster using the GS1_clust_FOM algorithm to select the variables used as input in pvclust used to perform hierarchical clustering. **S5 Fig. A: Ovalbumin serpin expression along the NAFLD progression.B: Positional gene enrichment.** A: Ovalbumin serpin expression along the NAFLD progression. MAT1A_15 and GNMT_ko8 are HCC mice samples where the serpins are overexpressed.B: Positional gene enrichment analysis using PGE program [46] shows that all the genes in ensemble chromosome band 6 p24.3 are overexpressed giving rise to the possibility a common mechanism of gene regulation. **S6 Fig. 91 human HCC data clustering.** Using complete hierarchical clustering using the Pearson correlation as a similarity measure it is possible to distinguish two stable clusters, cluster A and B that show statistical significant differences of survival length using by Kaplan-Meier plots and log-rank statistics analysis. **S7 Fig.** HNF4 alpha expression (log2 mouse KO vs wild type) in 3 and 8 month GNMT and MAT1A; and 15 month MAT1A (tumoral tissue, T). **S8 Fig.** Expression trend (log2 mouse KO vs wild type) of NAFLD progression genes regulated by HNF4a in 3 and 8 month GNMT and MAT1A; and 15 month MAT1A (tumoral tissue, T). **S9 Fig.** HNF4 alpha expression (log2 disease vs control) in human steatosis and NASH. **S10 Fig.** Expression trend (log2 mouse KO vs wild type) of NAFLD progression genes regulated by HNF4a in human steatosis and NASH. **S11 Fig.** Expression trend (log2 mouse KO vs wild type) of biosynthesis of unsaturated fatty acids in human steatosis and NASH; in 3, 8 month GNMT; and MAT1A KO mice and 15 month MAT1A tumors. **S12 Fig.** Expression (log2 mouse KO vs wild type) of stearoyl-CoA desaturase in human steatosis and NASH in 3, 8 month GNMT; and MAT1A KO mice and 15 month MAT1A tumors. **S13 Fig.** Expression trend (log2 mouse KO vs wild type) of phenylalanine, tyrosine and tryptophan biosynthesis in human steatosis and NASH; in 3, 8 month GNMT; and MAT1A KO mice and 15 month MAT1A tumors. **S14 Fig.** Expression trend (log2 mouse KO vs wild type) of androgen and estrogen metabolism in human steatosis and NASH; in 3, 8 month GNMT; and MAT1A KO mice and 15 month MAT1A tumors. **S15 Fig.** Expression trend (log2 mouse KO vs wild type) of arachidonic acid metabolism in human steatosis and NASH; in 3, 8 month GNMT; and MAT1A KO mice and 15 month MAT1A tumors. **S16 Fig.** Expression (log2 mouse KO vs wild type) of cyclooxygenase in human steatosis and NASH; in 3, 8 month GNMT; and MAT1A KO mice and 15 month MAT1A tumors. **S17 Fig.** Expression trend (log2 mouse KO vs wild type) of PPAR signaling pathway in human steatosis and NASH; in 3, 8 month GNMT; and MAT1A KO mice and 15 month MAT1A tumors. **S18 Fig.** Expression trend (log2 mouse KO vs wild type) of drug metabolism cytochrome P450 in human steatosis and NASH; in 3, 8 month GNMT; and MAT1A KO mice and 15 month MAT1A tumors. **S19 Fig.** Expression trend (log2 mouse KO vs wild type) of metabolism of xenobiotics by cytochrome P450 in human steatosis and NASH; in 3, 8 month GNMT; and MAT1A KO mice and 15 month MAT1A tumors. **S20 Fig.** Expression trend (log2 mouse KO vs wild type) of toll-like receptor signaling pathway in human steatosis and NASH; in 3, 8 month GNMT; and MAT1A KO mice and 15 month MAT1A tumors. **S21 Fig.** Expression trend (log2 mouse KO vs wild type) of p53 signaling pathway in human steatosis and NASH; in 3, 8 month GNMT; and MAT1A KO mice and 15 month MAT1A tumors. **S22 Fig.** Expression trend (log2 mouse KO vs wild type) of MAPK signaling pathway in human steatosis and NASH; in 3, 8 month GNMT; and MAT1A KO mice and 15 month MAT1A tumors. **S23 Fig.** Expression trend (log2 mouse KO vs wild type) of bile acid biosynthesis in 3, 8 month GNMT; and MAT1A KO mice and 15 month MAT1A tumors. **S1 Table. Summary of the most established biomarkers in NAFLD**. **S2 Table. Dunn and FOM indexes of the Signatures of NAFLD progression.** Dunn and FOM indexes of the Signatures of NAFLD progression resulting from the 14 different supervised clustering based feature selection methods on smoothed data; Ensemble error rate and stability in terms of Hamming distance of the Signatures of NAFLD progression resulting from the 7 different supervised clustering based feature selection methods that minimise the FOM index on smoothed data. **S3 Table. Enriched Transcription Factor binding sites.** Enriched Transcription Factor binding sites by means of Fisher exact test (p<0.05) in the signatures of NAFLD progression resulting from the two supervised clustering based feature selection methods which produced the optimal clustering result and the two ensemble signatures from raw and smoothed data. **S4 Table. Ensemble error rate and the number of the different feature selection methods used to build survival signature.** Ensemble error rate and the number of selected genes resulting from the different feature selection methods used to build the survival signatures common for human and mouse.
(DOCX)
