# Supplementary material for: Integrative Genomic Signatures Of Hepatocellular Carcinoma Derived from Nonalcoholic Fatty Liver Disease
Source: PLoS One. 2015 May 20;10(5):e0124544. doi: 10.1371/journal.pone.0124544 (PMC4439034; doi:10.1371/journal.pone.0124544)
Supplement: S1 File — Flow diagram of the steps performed by the RFE and RFE_MR method. Stage1: As it is a backwards procedure starts from the full matrix of selected genes. The process is iterative where the number of iterations either for first selection (x = number of selection iterations) or posterior refinement selection around the selection solution (y = number of refinement iterations) should be specified. It uses the class vector as input. Stage2: Evaluate the selected gene subset. Stage3a. If the process does not take into account the redundancy of the features (RFE): calculates the sample by sample MI excluding each gene. For each excluded gene defines a coefficient I as the difference of the sum of the sample by sample MI between classes and the sum of the sample by sample MI within groups. Stage 3b1: If the process takes into account the redundancy of the features (RFE_MR): for each gene calculates the average gene pairwise mutual information. Stage3b2: For each gene calculates the Coefficient II value by adding the average gene pairwise MI to the coefficient II. Stage4: Remove the m worst coefficient values and their corresponding genes and expression values. Stage5: Find the minimum error rate along the iterations and get the selected genes. S2 Fig. Flow diagram of the steps performed by the MRMR method. Stage1: As it is a forward search procedure, it starts from an empty set of selected genes. The process is iterative where the number of iterations should be specified and uses the class vector as input. Stage2: Calculate the normalized mutual information of the class vector with the vector containing each gene expression values along the samples. Stage3: a. For each gene calculate the average gene pairwise mutual information. b. For each gene in the subset of selected genes calculate the average gene pairwise mutual information. Stage4: For each gene define a coefficient value by dividing the value of the normalized mutual information with the average gene pairwise mutual [file pone.0124544.s001.docx]

**Supplementary Table S1.** Summary of the most established biomarkers in NAFLD.

| **Biomarker** | **Comment** | **Reference** |
| --- | --- | --- |
| **alanine aminotransferase** | The diagnosis is usually made when liver alanine aminotransferase (ALT) and/or aspartate aminotransferase (AST) and/or γ-glutamyl-transferase (GGT) enzymes are elevated. | [[1](#_ENREF_1)] , [[2](#_ENREF_2)] |
| **aspartate aminotransferase** |  | [[1](#_ENREF_1)] |
| **γ-glutamyl-transferase** |  | [[1](#_ENREF_1)] |
| **alpha-fetoprotein** | The most widely used serum HCC marker is alpha-fetoprotein. | [[3](#_ENREF_3)],[[4](#_ENREF_4)],[[5](#_ENREF_5)],[[6](#_ENREF_6)], [[7](#_ENREF_7)],[[8](#_ENREF_8)], [[9](#_ENREF_9)] |
| **TNFα** | There is an association between obesity and an increase in TNFα production  TNF-α inhibits the actions of adiponectin, its antagonist.  In hepatocytes excessive TNF-α and fatty acids but little adiponectin promotes lipid storage.  Increase in TNF-α within hepatocytes promotes hepatocyte oxidative stress and apoptosis that results in a recruitment of inflammatory cells from the immune system into the liver. | [[10](#_ENREF_10)],[[11](#_ENREF_11)],[[12](#_ENREF_12)],[[13](#_ENREF_13)] |
| **leptin** | Leptin induces factors that regulate the activity of profibrogenic cytokines. The signaling pathways induced by leptin include the JAK/STAT signaling pathway. The fibrogenic properties of leptin are related to the upregulation of procollagen mitogenesis and inhibition of hepatic stellate cell (HSC) apoptosis.  During chronic liver injury HSC activation plays a key role in fibrogenesis as become activated, transdifferentiated into myofibroblasts and produce excessive amount of Extracellular Matrix (ECM) and reactive oxigen species contributing to the oxidative stress and perpetuating the processes of chronic inflammation, lipid peroxidation, hepatocyte apoptosis, and fibrogenesis | [[10](#_ENREF_10)],[[14](#_ENREF_14)], [[15](#_ENREF_15)],[[16](#_ENREF_16)], [[17](#_ENREF_17)],[[18](#_ENREF_18)],[[19](#_ENREF_19)],[[20](#_ENREF_20)] |
| **Adiponectin** | Hepatocyte steatosis is promoted by reduced adiponectin activity which enhances fatty acid uptake, inhibits fatty acid oxidation and reduces lipid export. Adiponectin is also known to repress insulin resistance and prevent inflammation. | [[1](#_ENREF_1)] , [[10](#_ENREF_10)], [[21](#_ENREF_21)],[[22](#_ENREF_22)] |
| **TGF-β** | The extent of fibrosis that occurs during liver injury is dictated by this profibrogenic cytokine | [[10](#_ENREF_10)],[[16](#_ENREF_16)], [[23](#_ENREF_23)],[[24](#_ENREF_24)],[[25](#_ENREF_25)],[[26](#_ENREF_26)], [[27](#_ENREF_27)] |
| **NF-κB** | The retention of fatty acids releases signals that activate NF-κB within hepatocytes. This induces NF-κB-sensitive genes, increasing the generation of TNF-α, and IL-8. | [[10](#_ENREF_10)] |
| **IL-8** | This chemokine is one of the major mediators of the inflammatory response and in NAFLD recruits inflammatory cells from the immune system into the liver and promotes hepatocyte oxidative stress and eventual apoptosis. | [[10](#_ENREF_10)],[[28](#_ENREF_28)] |


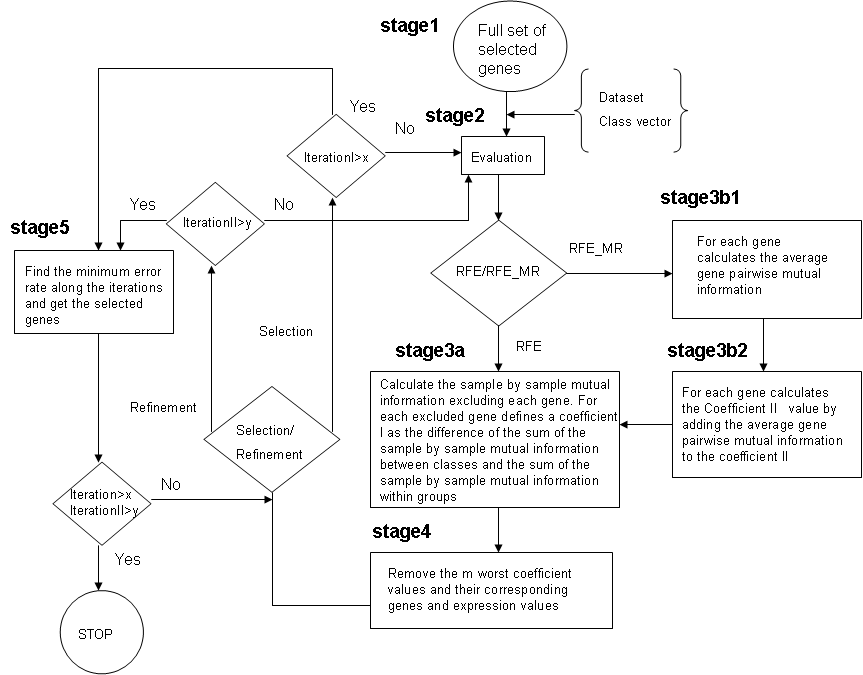


**Supplementary Figure S1.** Flow diagram of the steps performed by the RFE and RFE_MR method.

Stage1: As it is a backwards procedure starts from the full matrix of selected genes. The process is iterative where the number of iterations either for first selection (x=number of selection iterations) or posterior refinement selection around the selection solution (y= number of refinement iterations) should be specified. It uses the class vector as input.

Stage2: Evaluate the selected gene subset

Stage3a. If the process does not take into account the redundancy of the features (RFE): calculates the sample by sample MI excluding each gene. For each excluded gene defines a coefficient I as the difference of the sum of the sample by sample MI between classes and the sum of the sample by sample MI within groups.

Stage 3b1: If the process takes into account the redundancy of the features (RFE_MR): for each gene calculates the average gene pairwise mutual information

Stage3b2: For each gene calculates the Coefficient II value by adding the average gene pairwise MI to the coefficient II.

Stage4: Remove the m worst coefficient values and their corresponding genes and expression values. Stage5: Find the minimum error rate along the iterations and get the selected genes.


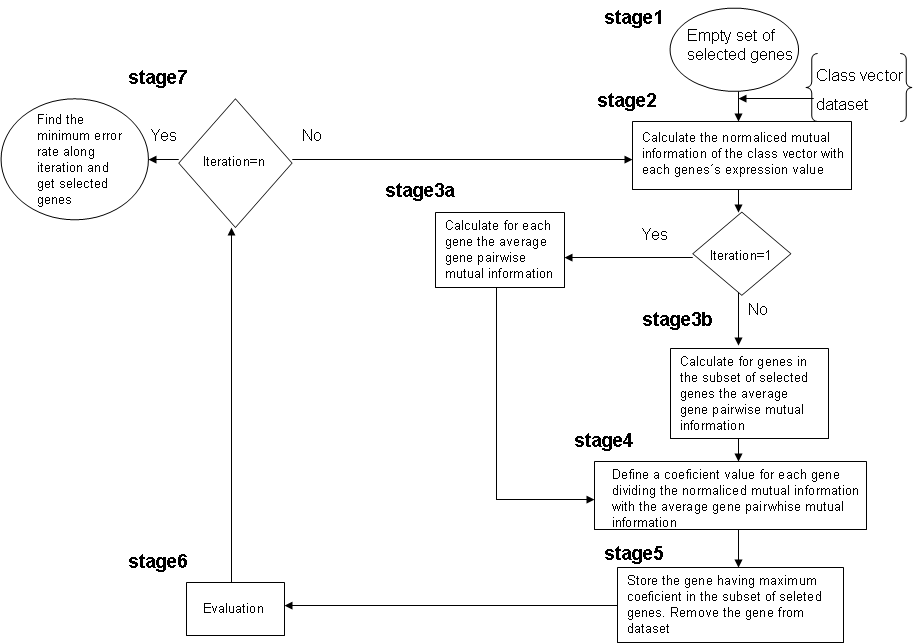


**Supplementary Figure S2.** Flow diagram of the steps performed by the MRMR method.

Stage1: As it is a forward search procedure, it starts from an empty set of selected genes. The process is iterative where the number of iterations should be specified and uses the class vector as input.

Stage2: Calculate the normalized mutual information of the class vector with the vector containing each gene expression values along the samples.

Stage3: a. For each gene calculate the average gene pairwise mutual information. b. For each gene in the subset of selected genes calculate the average gene pairwise mutual information.

Stage4: For each gene define a coefficient value by dividing the value of the normalized mutual information with the average gene pairwise mutual information.

Stage5: Store the gene having the maximum coefficient value and remove from the matrix the corresponding gene.

Stage6: Evaluate.

Stage7: Find the minimum error rate along the iterations and get selected genes.


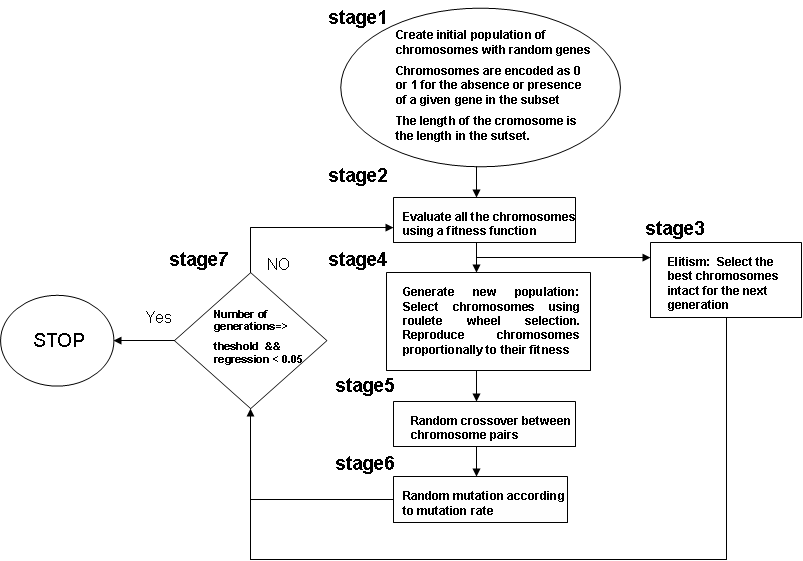


**Supplementary Figure S3.** Flow diagram of the GA procedure.

Stage 1: The procedure initially creates a number of random variable sets (chromosomes). These variable sets form a population of chromosomes. Each random set is created with an initialization that randomly selects 70 genes from the total 504.

Stage 2: Each chromosome in the population is evaluated for its ability to predict the group membership of each sample in the dataset (fitness function).

Stage 3: Elitism: select the fittest individual intact for the next generation.

Stage 4: The population of chromosomes is replicated. The roulette wheel selection ensures that chromosomes with a higher fitness score will generate a more numerous offspring.

Stage 5: The genetic information contained in the replicated parent chromosomes is combined through genetic crossover with a crossover probability (For the parameters see supplementary table 4 and “*Parameters in the Genetic Algorithm*” supplementary section). The chromosomes are ranked according to their fitness value. Above the crossover probability the best chromosomes are maintained intact for the next generation. Below the crossover probability two randomly selected parent chromosomes are used to create two new chromosomes. This crossover mechanism allows a better exploration of possible solutions recombining good chromosomes.

Stage 6: Mutations are then introduced in the new chromosomes generated by crossover randomly with a mutation probability. These mutations produce that new genes are used in chromosomes.

Stage 7: The process is repeated from stage 2 until the number of generations exceeds certain threshold (100) and the regression between the population of chromosome’s minimum error rate and the generation is less than 0.05. The cycle of replication (stage 3), genetic crossover (stage 4) and mutations (stage 5) is called generation.

A)

B)


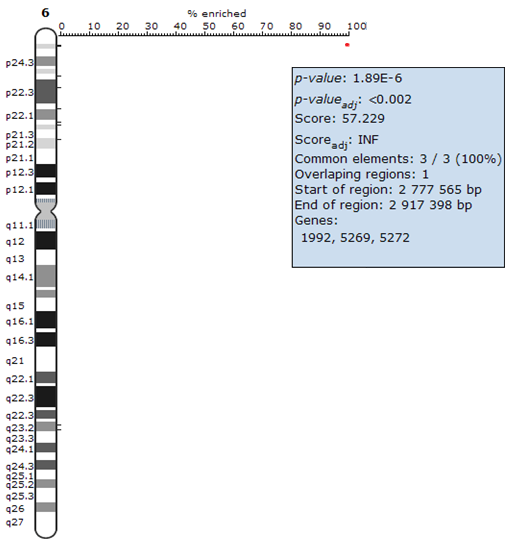


**Supplementary Figure S4.** A: Ovalbumin serpin expression along the NAFLD progression. MAT1A_15 and GNMT_ko8 are HCC mice samples where the serpins are overexpressed. B: Positional gene enrichment analysis using PGE program [[29](#_ENREF_29)] shows that all the genes in ensemble chromosome band 6 p24.3 are overexpressed giving rise to the possibility a common mechanism of gene regulation.


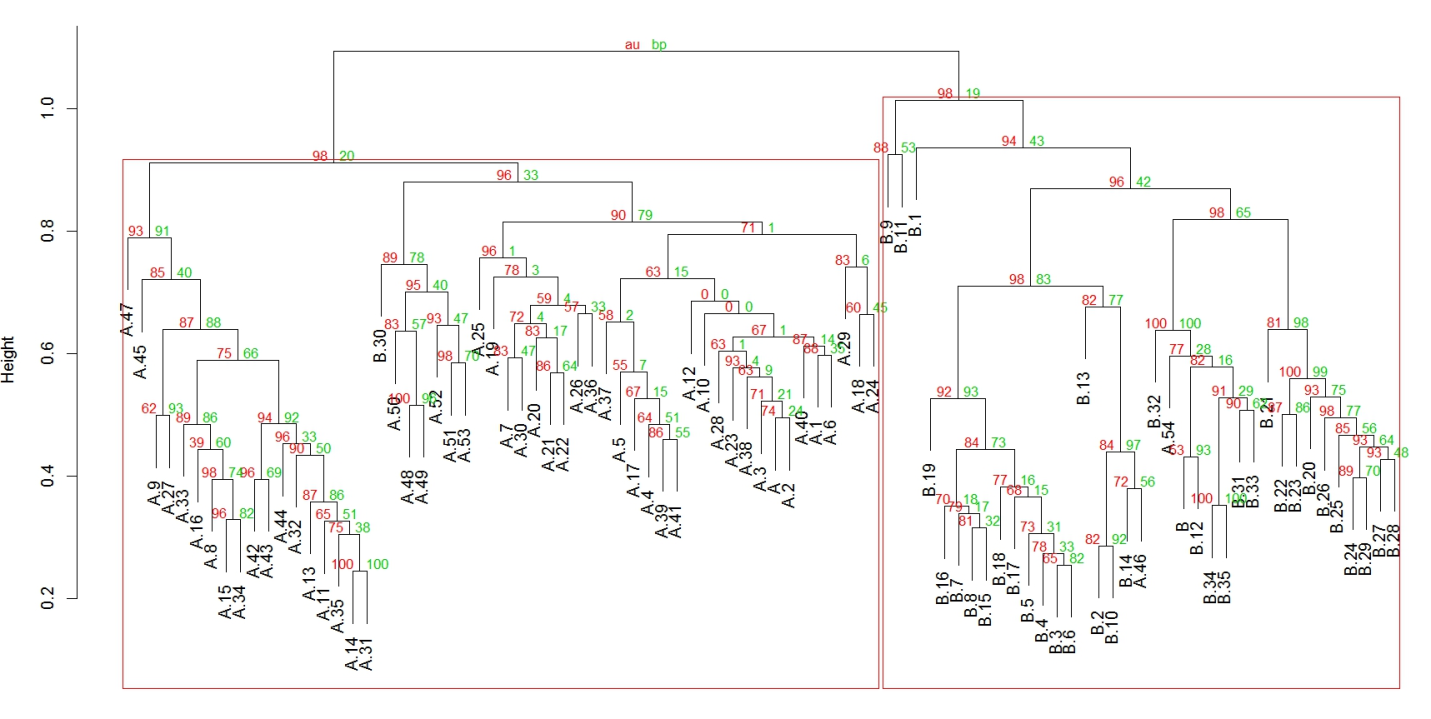


**Supplementary Figure S5.** 91 human HCC data clustering. Using complete hierarchical clustering using the Pearson correlation as a similarity measure it is possible to distinguish two stable clusters, cluster A and B that show statistical significant differences of survival length using by Kaplan-Meier plots and log-rank statistics analysis.


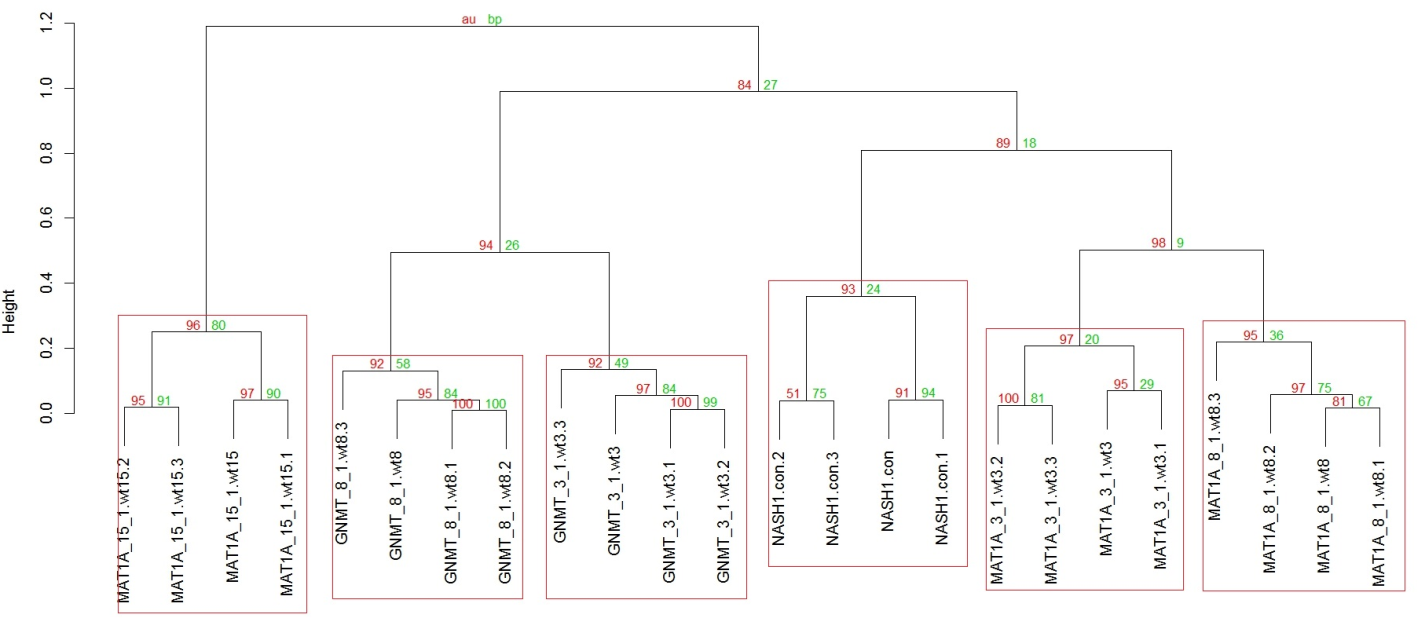


**Supplementary Figure S6.** Tree structure where each of the stages of the disease has been clustered in a single cluster using the GS1_clust_FOM algorithm to select the variables used as imput in pvclust used to perform hierarchical clustering.

**Supplementary Figure S7:** HNF4 alpha expression (log2 mouse KO vs wild type) in 3 and 8 month GNMT and MAT1A; and 15 month MAT1A (tumoral tissue, T).

**Supplementary Figure S8:** Expression trend (log2 mouse KO vs wild type) of NAFLD progression genes regulated by HNF4a in 3 and 8 month GNMT and MAT1A; and 15 month MAT1A (tumoral tissue, T).

**Supplementary Figure S9:** HNF4 alpha expression (log2 disease vs control) in human steatosis and NASH.

**Supplementary Figure S10:** Expression trend (log2 mouse KO vs wild type) of NAFLD progression genes regulated by HNF4a in human steatosis and NASH.

Supplementary Table S2. Enriched Transcription Factor binding sites by means of Fisher exact test (p<0.05) in the signatures of NAFLD progression resulting from the two supervised clustering based feature selection methods which produced the optimal clustering result and the two ensemble signatures from raw and smoothed data.

| RFE_clust_Dun | GS1_clust_Fom | ensemble | Smoothed-ensemble |
| --- | --- | --- | --- |
| NR2F1 | RELA | Nkx2-5 | HNF4A |
| HNF4A |  | FOXF2 |  |
|  |  | HLF |  |
|  |  | Fos |  |
|  |  | HNF4A |  |
|  |  | Nobox |  |
|  |  | Cebpa |  |
|  |  | Pdx1 |  |
|  |  | Gfi |  |

**Supplementary Table S3.** Ensemble error rate and the number of selected genes resulting from the different feature selection methods used to build the survival signatures common for human and mouse.

| **METHOD** | **GENES** | **Hamming distance** | **Ensemble correctly classified instances** |
| --- | --- | --- | --- |
| **GS1** | 9 | 8.149 | 88 |
| **GS2** | 9 | 7.792 | 88 |
| **F-TEST** | 12 | 4.853 | 90 |
| **RFE_SVM** | 24 | 1.416 | 93 |
| **MRMR** | 10 | 5.103 | 90 |

**Supplementary Table S4.** Dunn and FOM indexes of the Signatures of NAFLD progression resulting from the 14 different supervised clustering based feature selection methods on smoothed data; Ensemble error rate and stability in terms of Hamming distance of the Signatures of NAFLD progression resulting from the 7 different supervised clustering based feature selection methods that minimise the FOM index on smoothed data.

| Signatures of NAFLD  progression Smoothed | **Method** | **Dunn** | **FOM** | **Ensemble_error FOM** | **NAHD** |
| --- | --- | --- | --- | --- | --- |
|  | **GS1_Clust** | 0.515 | 0.0007 | 0 | 7.510 |
|  | **GS2_Clust** | 0.747 | 0.0010 | 0 | 4.425 |
|  | **F-TEST_Clust** | 0.664 | 0.0019 | 0.042 | 2.574 |
|  | **RFE_Clust** | 0.662 | 0.0007 | 0.083 | 7.217 |
|  | **RFE_MR_Clust** | 0.681 | 0.0015 | 0.083 | 2.131 |
|  | **GA_Clust** | 0.668 | 0.0007 | 0 | 6.951 |
|  | **MRMR_Clust** | 0,709 | 0.0007 | 0.291 | 3.697 |

**Supplementary Table S5***:* The minimum error rates of the different combination of parameters used to perform the parameter tuning

| **Population size** | **Crossover probability** | **Mutation probability** | **Number of minimum generations** | **Minimum error rate** | **Number of genes** |
| --- | --- | --- | --- | --- | --- |
| 100 | 0.7 | 0.001 | 300 | 0.065 | 242 |
| 100 | 0.7 | 0.01 | 300 | 0.06 | 243 |
| 100 | 0.7 1^st^ 160 generations-0.9 from 160-300 generations | 0.01 1^st^ 160 generations-0.1 from 160-300 generations | 300 | 0.064 | 241 |
| 100 nonrandom initial population | 0.7 1^st^ 160 generations-0.9 from 160-300 generations | 0.01 1^st^ 160 generations-0.1 from 160-300 generations | 300 | 0.056 | 153 |

**Supporting Methods: *Parameters in the Genetic Algorithm***

Several aspects from the GAs have been determined when they are used to this specific feature selection problem: the initial population of chromosomes has been generated either with random genes or randomly selecting 70 genes for each chromosome what we called nonrandom initial population. Each individual in the population represents a candidate solution to the feature selection problem. The codification of the chromosome it is represented by a binary vector of dimension m (where m is the total number of attributes)[[30](#_ENREF_30), [31](#_ENREF_31)]. If a bit is a 1, it means that the corresponding attribute is selected while a value of 0 indicates that the corresponding attribute is not selected. Hence, the length of the chromosome will be the number of features of the sample set. The population size is set initially to 100 [[32](#_ENREF_32)].

The most widely used standard genetic operators (one-point crossover and single point mutation) are implemented. Their probabilities are initially fixed to 0.7 and 0.00 1 respectively, as it is suggested in [[33](#_ENREF_33)]. In order to avoid the GA getting stuck in a local minimum we used an strategy consisting in incrementing the mutation rate and the crossover probability once reached the generations where it gets stuck in the local optimum. The fitness of an individual is determined by using the classification error produced by any of the proposed evaluation procedures as a measure of goodness for a particular feature subset.

This generational process is repeated until a termination condition has been reached. The termination condition is reached when a fixed number of generations take place (300) or the regression coefficient of classification performance over the generations is less than 0.05. Next, it comes the selection strategy, that is, how to select parents for recombination. We used the roulette wheel selection method, which ensures that the better chromosome will have better chance to be selected.

Suppose the fitness (error rate) of N individuals in a population are: f_1_, f_2_, f_3_,..., f_N_.

In roulette wheel selection, the probability of an individual being selected is

P_i_=f_i_/ (f_1_+f_2_+...+ f_N_)

As we are dealing with error rates, we want to inverse the probabilities to ensure that the better chromosome will have better chance to be selected:

Pinverse=1- P_i_

$total=\sum_{i=n} P_{i}$ Where n is the number of chromosomes.

Then a parent is selected by going through the following steps:

a. Generate a random value r

between 0 and total.

b. Set sum=0;

c. for i=1 to N do

begin

sum=sum+ P_i_;

if (sum>=r)

return i;

end

For parameter tuning we have ran the GA with different combination of the parameters in Supplementary Table S4.

**Supplementary Figure S11:** Expression trend (log2 mouse KO vs wild type) of biosynthesis of unsaturated fatty acids in human steatosis and NASH; in 3, 8 month GNMT; and MAT1A KO mice and 15 month MAT1A tumors.

**Supplementary Figure S12:** Expression (log2 mouse KO vs wild type) of stearoyl-CoA desaturase in human steatosis and NASH; in 3, 8 month GNMT; and MAT1A KO mice and 15 month MAT1A tumors.

**Supplementary Figure S13:** Expression trend (log2 mouse KO vs wild type) of phenylalanine, tyrosine and tryptophan biosynthesis in human steatosis and NASH; in 3, 8 month GNMT; and MAT1A KO mice and 15 month MAT1A tumors.

**Supplementary Figure S14:** Expression trend (log2 mouse KO vs wild type) of androgen and estrogen metabolism in human steatosis and NASH; in 3, 8 month GNMT; and MAT1A KO mice and 15 month MAT1A tumors.

**Supplementary Figure S15:** Expression trend (log2 mouse KO vs wild type) of arachidonic acid metabolism in human steatosis and NASH; in 3, 8 month GNMT; and MAT1A KO mice and 15 month MAT1A tumors.

**Supplementary Figure S16:** Expression (log2 mouse KO vs wild type) of cyclooxygenase in human steatosis and NASH; in 3, 8 month GNMT; and MAT1A KO mice and 15 month MAT1A tumors.

**Supplementary Figure S17:** Expression trend (log2 mouse KO vs wild type) of PPAR signaling pathway in human steatosis and NASH; in 3, 8 month GNMT; and MAT1A KO mice and 15 month MAT1A tumors.

**Supplementary Figure S18:** Expression trend (log2 mouse KO vs wild type) of drug metabolism cytochrome P450 in human steatosis and NASH; in 3, 8 month GNMT; and MAT1A KO mice and 15 month MAT1A tumors.

**Supplementary Figure S19:** Expression trend (log2 mouse KO vs wild type) of metabolism of xenobiotics by cytochrome P450 in human steatosis and NASH; in 3, 8 month GNMT; and MAT1A KO mice and 15 month MAT1A tumors.

**Supplementary Figure S20:** Expression trend (log2 mouse KO vs wild type) of toll-like receptor signaling pathway in human steatosis and NASH; in 3, 8 month GNMT; and MAT1A KO mice and 15 month MAT1A tumors.

**Supplementary Figure S21:** Expression trend (log2 mouse KO vs wild type) of p53 signaling pathway in human steatosis and NASH; in 3, 8 month GNMT; and MAT1A KO mice and 15 month MAT1A tumors.

**Supplementary Figure S22:** Expression trend (log2 mouse KO vs wild type) of MAPK signaling pathway in human steatosis and NASH; in 3, 8 month GNMT; and MAT1A KO mice and 15 month MAT1A tumors.

**Supplementary Figure S23:** Expression trend (log2 mouse KO vs wild type) of bile acid biosynthesis in 3, 8 month GNMT; and MAT1A KO mice and 15 month MAT1A tumors.

REFERENCES:

1. Jiang J, Torok N. Nonalcoholic Steatohepatitis and the metabolic syndrome. Metabolic syndrome and related disorders. 2008;6(1).

2. Chen CH, Huang MH, Yang JC, Nien CK, Yang CC, Yeh YH, et al. Prevalence and risk factors of nonalcoholic fatty liver disease in an adult population of Taiwan: metabolic significance of nonalcoholic fatty liver disease in nonobese adults. Clinical Journal of Gastroenterology. 2006 40.

3. Beale G, Chattopadhyay D, Gray J, Stewart S, Hudson M, Day C, et al. AFP, PIVKAII, GP3, SCCA-1 and follisatin as surveillance biomarkers for hepatocellular cancer in non-alcoholic and alcoholic fatty liver disease. BMC Cancer 2008 8:200.

4. Trevisani F, D'Intino PE, Morselli-Labate AM, Mazzella G, Accogli E, Caraceni P, et al. Serum alpha-fetoprotein for diagnosis of hepatocellular carcinoma in patients with chronic liver disease: influence of HBsAg and anti-HCV status. Journal of Hepatology. 2001;34.

5. Kew MC. Alpha-fetoprotein. . In: Read AE, editor. Modern trends in gastroenterology: Butterworths, London; 1975.

6. Ishii M, Gama H, Chida N, Ueno Y, Shinzawa H, Takagi T, et al. Simultaneous measurements of serum alpha-fetoprotein and protein induced by vitamin K absence for detecting hepatocellular carcinoma. The American Journal of Gastroenterology. 2000;95.

7. Liaw YF, Tai DI, Chu CM, Lin DY, Sheen IS, Chen TJ, et al. Early detection of hepatocellular carcinoma in patients with chronic type B hepatitis. A prospective study. . Gastroenterology. 1986 90.

8. Sherman M, Peltekian KM, Lee C. Screening for hepatocellular carcinoma in chronic carriers of hepatitis B virus: incidence and prevalence of hepatocellular carcinoma in a North American urban population. . Hepatology 1995 22.

9. Zhang B, Yang B. Combined alpha fetoprotein testing and ultrasonography as a screening test for primary liver cancer. Journal of Medical Screening. 1999;6.

10. Ionel Copaci, Laurentiu Micu, Voiculescu M. The Role of Cytokines in Non-Alcoholic Steatohepatitis. A Systematic Review. J Gastrointestin Liver Dis. 2006 15(4):363-73.

11. Kern PA, Saghizadeh M, Ong JM, Bosch RJ, Deem R, Simsolo RB. The expression of tumor necrosis factor in human adipose tissue. Regulation by obesity, weight loss, and relationship to lipoprotein lipase. . The Journal of Clinical Investigation. 1995;95.

12. Crespo J, Cayon A, Fernandez-Gil P. Gene expression of tumor necrosis factor alpha and TNF-receptors p55 and p75, in non-alcoholic steatohepatitis patients. Hepatology 2001;34.

13. Valenti L, Fracanzani AL, Dongiovanni P. Tumor necrosis factor alpha promoter polymorphisms and insulin resistance in nonalcoholic fatty liver disease. Gastroenterology 2002.

14. Niswender KD, Schwartz MW. Insulin and leptin revisited: adiposity signals with overlapping physiological and intracellular signaling capabilities. . Frontiers in Neuroendocrinology. 2003;24.

15. Minokoshi Y, Kim YB, Peroni OD. Leptin stimulates fatty-acid oxidation by activating AMP-activated protein kinase. Nature 2002;415.

16. Leclercq IA, Farrell GC, Schriemer R, Robertson GR. Leptin is essential for the hepatic fibrogenic response to chronic liver injury. Journal of Hepatology. 2002;37.

17. Wolf G, Hamann A, Han DC, Thaiss F, Ziyadeh FN, Stahk RA. Leptin stimulates proliferation and TGF-beta expression in renal glomerular endothelial cells: potential role in glomerulosclerosis. Kidney International. 1999;56

18. Frank S, Stallmeyer B, Kampfer H, Kolb N, Pfeilschifter J. Leptin enhances wound re-epithelialization and constitutes a direct function of leptin in skin repair. The Journal of Clinical Investigation. 2000;106

19. Ring BD, Scully S, Davis CR, Baker MB, Cullen MJ, Pelleymounter MA. Systemically and topically administered leptin both accelerate wound healing in diabetic ob/ob mice. Endocrinology. 2000;141

20. Ikejima K, Honda H, Yoshikawa M, Hirose M, Kitamura T, Takei Y. Leptin augments inflammatory and profibrogenic responses in the murine liver induced by hepatotoxic chemicals. Hepatology. 2001;34

21. Kaser S, Moschen A, Cayon A, Kaser A. Adiponectin and its receptors in nonalcoholic steatohepatitis. Gut 2005;54.

22. Musso G, Gambino R, Biroli G, Carello M. Hypoadiponectinemia predicts the severity of hepatic fibrosis and pancreatic β-cell dysfunction in nondiabetic nonobese patients with nonalcoholic steatohepatitis. American Journal of Gastroenterology. 2005;100.

23. Friedman SL. Cytokines and fibrogenesis. Seminars in Liver Disease. 1999;19

24. Hellebrand C, Stefanovic B, Giordano F, Burchardt ER, Brenner DA. Role of TGFβ1 in initiating hepatic stellate cell activation in vivo. Journal of Hepatology. 1999;30.

25. George J, Roulot D, Koteliansky VE, Bissel DM. In vivo inhibition of rat stellate cell activation by soluble transforming growth factor β type II receptor: a potential new therapy for hepatic fibrosis. Proceedings of the National Academy of Sciences of the United States of America. 1999;96

26. Qi Z, Atsuchi N, Ooshima A, Takeshita A, Ueno H. Blockade of type β transforming growth factor signaling prevents liver fibrosis and dysfunction in the rat. Proceedings of the National Academy of Sciences of the United States of America. 1999;96

27. Sanderson N, Factor V, Nagy P, Kopp J, Kondaiah P, Wakefield L. Hepatic expression of mature transforming growth factor beta 1 in transgenic mice results in multiple tissue lesions. Proceedings of the National Academy of Sciences of the United States of America. 1995;92.

28. Bruun JM, Lihn AS, Verdich C, Pedersen SB, Toubro S, Astrup A, et al. Regulation of adiponectin by adipose tissue-derived cytokines: in vivo and in vitro investigations in humans. . American Journal of Physiology - Endocrinology and Metabolism 2003;285.

29. De Preter K, Barriot R, Speleman F, Vandesompele J, Moreau Y. Positional gene enrichment analysis of gene sets for high-resolution identification of overrepresented chromosomal regions. Nucleic Acids Res. 2008;36(7):43.

30. Lanzi PL. Fast feature selection with genetic algorithms: a filter approach. IEEE International Conference on Evolutionary Computation; Indianapolis, IN, USA1997. p. 537-40.

31. Yang J, Honavar, V. Feature subset selection using a genetic algorithm. IEEE Intelligent Systems. 1998;13:44 - 9.

32. Martin-Bautista MJ, Vila, M.A. A survey of genetic feature selection in mining issues. Congress on Evolutionary Computation, CEC 99 1999. p. 1321.

33. Mitchell M. An Introduction to Genetic Algorithms. Cambridge, MA: MIT Press.; 1996.
